# Sustainable Remediation of Pharmaceuticals Using Crop-Residue-Derived Carbons: Bridging Multi-Component Adsorption and DFT Perspectives

**DOI:** 10.3390/molecules31071162

**Published:** 2026-03-31

**Authors:** Assel A. Kurtebayeva, Silvia Álvarez-Torrellas, Juan García, Helder T. Gomes, Juan M. Garrido-Zoido, Maria Victoria Gil, Seitzhan A. Orynbayev, Marzhan S. Kalmakhanova

**Affiliations:** 1 Department of Chemistry and Chemical Technology, M. Kh. Dulaty Taraz University, Taraz 080012, Kazakhstan; d.a.kurtebayeva@dulaty.kz (A.A.K.);; 2 Catalysis and Separation Processes Group, Chemical Engineering and Materials Department, Faculty of Chemistry, Complutense University, Avda. Complutense s/n, 28040 Madrid, Spain; jgarciar@ucm.es; 3 Centro de Investigação de Montanha (CIMO), LA SusTEC, Instituto Politécnico de Bragança, Campus de Santa Apolónia, 5300-253 Bragança, Portugal; 4 IACYS-Green Chemistry and Sustainable Development Unit, Department of Organic and Inorganic Chemistry, Faculty of Sciences, University of Extremadura, 06006 Badajoz, Spain; jmgarridoz@unex.es (J.M.G.-Z.); vgil@unex.es (M.V.G.)

**Keywords:** activated carbon, adsorption, agro-industrial waste, chemical activation, emerging contaminants

## Abstract

This work is devoted to the synthesis and comprehensive study of activated carbons (ACs) obtained from agricultural wastes—specifically corn cob (C) and onion (O)—for the effective removal of paracetamol (PCM) and sulfamethoxazole (SMX) from aqueous media. The synthesis was carried out by chemical activation using H_3_PO_4_, HNO_3_, and NaOH as activating agents, which made it possible to obtain materials with a clearly defined microporous structure (microporous fraction V_micro_/V_total_ = 0.75–0.81) and specific surface chemistry. Particular attention was paid to studying the kinetics and equilibrium of adsorption in both single-component and binary (two-pollutant) systems. It was established that the equilibrium time is 8 h, and the experimental data are best described by a pseudo-second-order kinetic model. During binary adsorption tests, the competitive behavior was observed for certain materials, such as the corn-derived carbon activated with HNO_3_ (AC-CN) and the onion-derived carbon activated with HNO_3_ (AC-ON), where molecules compete for active sites. Conversely, synergistic effects were identified in other systems, controlled by specific surface-functional groups and hydration effects. The maximum adsorption capacity was found to be 29.4 mg∙g^−1^ for PCM on the AC-CN sample. Adsorption mechanisms, including multilayer isotherm profiles and the competition between pollutant and water molecules, were interpreted using quantum chemical calculations within the framework of Density Functional Theory (DFT). These calculations revealed that partial deprotonation and intense solvation of SMX molecules at natural pH reduce their adsorption capacity. In contrast, the PCM structure favors π-π interactions and the formation of strong hydrogen bonds with oxygen-containing groups on the carbon surface. These results demonstrate the high potential of using agro-industrial waste to create a new generation of selective adsorbents with tailored surface properties.

## 1. Introduction

The quality of water resources—for both drinking and irrigation—remains a critical global concern due to increasing anthropogenic impacts and continuous pollutant discharges [1,2,3]. Over the past decades, “emerging contaminants” (ECs), including pesticides, personal care products, PFAS, and pharmaceuticals, have attracted significant scientific attention [3,4,5,6,7,8]. Since a universally approved regulatory list is still evolving [9], these pollutants are continuously detected in effluents at trace levels (µg/L to ng/L). Due to their poor biodegradability, they often escape conventional biological wastewater treatment plants (WWTPs), eventually accumulating in drinking water bodies and threatening both ecosystems and human health [10].

Pharmaceutical compounds, in particular, are increasingly detected in surface and groundwater worldwide [11,12,13]. Tighter regulations and the implementation of advanced treatment technologies are crucial, especially in water-scarce regions like Central Asia, where wastewater reuse is an essential water security strategy [14,15]. Originating from biological excretions, improper disposal, and medical facilities [16,17], these pharmaceutical residues persist through conventional treatments [18,19]. Furthermore, real-world mixtures of these compounds pose amplified environmental risks due to synergistic ecotoxicological effects [12,13,19].

Because conventional methods, such as coagulation and chlorination, cannot ensure complete pollutant removal [18], advanced solutions like adsorption have been widely adopted due to their reliability, low energy consumption, and cost-effectiveness [20,21,22,23,24]. Among various adsorbents [25,26,27,28], activated carbons (ACs) are highly preferred for their extensive specific surface area and tunable porosity [23,29,30,31,32,33]. Recently, the production of ACs has shifted toward utilizing biodegradable agro-industrial wastes—such as rice husks [34], onion husks [35], apricot seeds [36], coconut shells [37], and other biomass [38,39]. This approach not only provides economically advantageous and environmentally sound materials for wastewater treatment but also significantly contributes to sustainable waste management under circular economy principles (Table 1).

Typically, ACs are synthesized via physical activation (using steam or CO_2_ at 700– 1000 °C) [40,41,42,43] or chemical activation, which operates at lower temperatures (400–700 °C) and yields moderate-to-high surface areas with an optimal mesoporosity ratio [41,44,45,46,47,48,49]. Chemical activation with specific acids and bases is key to precisely adjusting porosity and surface chemistry. For instance, phosphoric acid (H_3_PO_4_) promotes one-step carbonization and pore development, introducing acidic oxygen/phosphorus groups (C–O–P) with a lower environmental impact than ZnCl_2_ [50,51,52]. Conversely, sodium hydroxide (NaOH) acts as a strong alkaline activator that generates remarkable microporosity and excellent capacity for large pollutant molecules [53,54]. Post-treatment with nitric acid (HNO3) is often employed to functionalize the formed carbons, sharply increasing surface acidity by enriching them with carboxyl, lactone, and phenolic groups, despite a potential slight reduction in specific surface area [55,56].

To evaluate the specific reactivity of the engineered ACs (AC-ON and AC-CN), paracetamol (PCM) and sulfamethoxazole (SMX) were selected as probe molecules. Their critical role as ubiquitous priority pollutants [57,58], combined with their high solubility and resistance to traditional treatments, poses serious environmental risks [59,60]. Choosing these pharmaceuticals over well-studied dyes or heavy metals is justified by their complex aromatic structures and “emerging” status [61,62]. Unlike heavy metals, which rely heavily on ion exchange mechanisms [63,64], PCM and SMX adsorption involves complex interactions such as π-π stacking and hydrogen bonding, allowing for a comprehensive assessment of the carbon’s surface chemical potential [65,66].

**Table 1:** Comparative performance of AC-ON and AC-CN against previously reported activated carbons for the removal of pharmaceutical contaminants.

Adsorbent	Target Pollutants and Matrix	*q*_max_ (mg·g^−1^) and Removal (%)	Key Operating Conditions (pH, T, Dose)	Regeneration/Stability	Main Advantages vs. AC-ON/AC-CN	Citations
AC-ON	paracetamol (PCM) and sulfamethoxazole (SMX)	PCM: 23.9 mg/g; SMX: 11.0 mg/g. Removal up to 92%	pH = neutral; T = room temperature °C; dose = 2.5 g·L^−1^	Not yet investigated	Sustainable onion waste precursor; demonstrates unique cooperative adsorption in binary systems (increasing capacity by ~70% for SMX).	In this work
AC-CN	paracetamol (PCM) and sulfamethoxazole (SMX)	PCM: 29.4 mg/g; SMX: 20.3 mg/g. Removal up to 99%	pH = neutral; T = room temperature °C; dose = 2.5 g·L^−1^	Not yet investigated	Superior performance for SMX (~84% higher than AC-ON); well-developed microporosity (V_micro_ = 0.182 cm^3^/g) and higher S_BET_	In this work
AC (commercial or biomass-derived)	Mixed heavy metals (Ni^2+^, Mn^2+^, Cr^6+^, Cd^2+^)	*q*_max_ (Ni^2+^) ≈ 3.98; Cd^2+^ ≈ 1.04; Mn^2+^ ≈ 1.05 mg g^−1^; Cr^6+^ mainly physisorbed; 66–70% removal at best	pH ≈ 6; 25 °C; dose 2.5–100 g L^−1^	No specific regeneration enhancement; Mn^2+^ removal very poor	Reference for unmodified AC in competitive systems; shows limitations for Cr^6+^ selectivity and Mn^2+^ uptake	[67]
HNO_3_-oxidized AC (AGC/APC)	Same mixed metals as above	For Cr^6+^: *q*_max_ ≈ 10.5–12.1 mg·g^−1^; ≈100% removal; Ni^2+^, Cd^2+^, Mn^2+^ removal decreased by 23–37% vs. AC	pH ≈ 6; 25 °C; dose 2.5–100 g L^−1^	Good structural stability; surface becomes more hydrophilic	Demonstrates strong selectivity to Cr^6+^ in multimetal systems via ion-exchange on acidic groups	[67]
ZnO/Fe_2_O_3_/AC	Cyanide in wastewater	*q*_max_ ≈ 101 mg·g^−1^; 82.5% removal; improved vs. bare AC (78.1 mg·g^−1^; 66.3%)	Optimized pH; dose not high; Langmuir isotherm (0.56 < R_L < 0.64)	Modified ACs more reusable and stable than bare AC	High capacity for CN^−^; shows benefit of AC–oxide nanocomposites in anion removal	[68]
ACS film (AC-based)	Cr(VI) in water; textile effluent	*q*_max_ ≈ 467 mg·g^−1^ for Cr(VI); Freundlich isotherm; spontaneous, endothermic	RSM-optimized pH, dose, contact time	Maintains high efficiency over 5 cycles	Very high capacity, dual removal (Cr(VI) + SDS); benchmark for high-end AC-based Cr(VI) adsorbents	[69]
g-C_3_N_4_-coated AC (AC-CN@US)	Organic pollutant (ciprofloxacin)	High adsorption (~50% in 2 h) plus enhanced degradation; surface area ≈ 329 m^2^·g^−1^ (vs. 115 m^2^ g^−1^ for ball-milled composite)	pH~7; 25 mg in 100 mL of 30 mg·L^−1^; solar light	Stable over ≥5 cycles; g-C_3_N_4_ firmly fixed; no significant leaching	Ultrasonic route gives best dispersion and capacity; low-cost biomass-derived AC; representative of high-performance AC–g-C_3_N_4_	[70]

Despite extensive research on biomass-derived ACs, several gaps remain. First, most studies focus on single-component systems, whereas real wastewater contains mixtures whose competitive interactions remain underexplored. Second, comparative analyses linking specific chemical activation methods (such as HNO_3_ functionalization) to adsorption selectivity in binary PCM/SMX systems are fragmentary. Finally, there is a lack of studies combining experimental data on specific agro-wastes (onion husks and corn cobs) with theoretical Density Functional Theory (DFT) calculations to elucidate the energetic mechanisms of adsorbate-adsorbent interactions.

In this study, several carbon materials will be synthesized by chemical activation using onion husks and corn cob as agricultural waste precursors. The obtained activated carbons will be used as adsorbents for the efficient removal of two pharmaceutical compounds of emerging concern, e.g., paracetamol (PCM), and sulfamethoxazole (SMX), from aqueous solution. Therefore, the main objectives of this work are as follows: (i) to characterize the textural, surface chemical and morphological properties of the carbon materials; (ii) to investigate the adsorptive removal of PCM and SMX by using the obtained activated carbons—kinetic and equilibrium adsorption studies; (iii) to study the competitive or synergistic effect on the adsorption of binary (PCM-SMX) solutions; (iv) to explore the adsorption performance of the carbon for the treatment of a WWTP effluent as a proof of concept; and (v) to elucidate PCM and SMX adsorption mechanism by using theoretical DFT calculations.

This study contributes to the achievement of the UN Sustainable Development Goals (SDG 6 and SDG 12) by proposing a solution to the problem of removing pharmaceuticals from water using sorbents derived from renewable plant-based raw materials. This addresses global challenges related to environmental safety and the transition to a circular economy.

## 2. Results

### 2.1. Characterization of the Activated Carbons

#### 2.1.1. Textural Properties of the Activated Carbons

**Figure 1:**
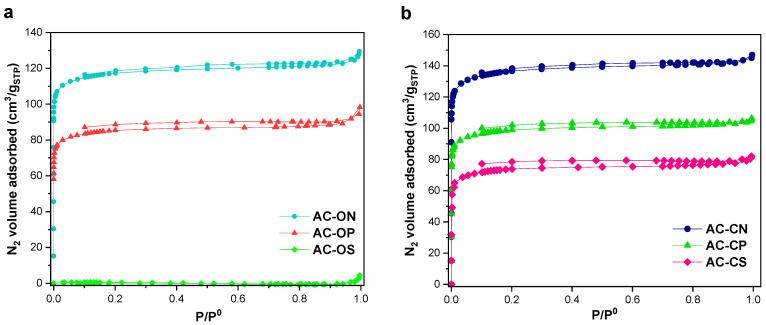
N_2_ adsorption–desorption isotherms at −196 °C of the activated carbons obtained from (**a**) onion waste (**b**) corn cob.

The N_2_ adsorption–desorption isotherms of the synthesized materials can be seen in Figure 1, and the corresponding textural properties can be found in Table 2. The pore size distribution of the ACs is shown in Figure S1 (Supplementary Materials). From the results, it can be observed that the obtained activated carbons were characterized by a moderate specific surface area (SBET = 251–462 m^2^∙g^−1^), which is consistent with the results of other works for activated carbons synthesized from strawberry seeds and pistachio shells [71].

Also, activated carbons showed V_Total_ of 0.125–0.224 cm^3^∙g^−1^, with micropore volumes of 0.094–0.182 cm^3^∙g^−1^ (75–81% of the total pore volume). According to IUPAC classification, N_2_ adsorption–desorption isotherms can be classified as Type-Ia, typical of microporous materials. Similar behavior was found in the work of Blachnio et al. 2020 [72,73], where highly microporous activated carbons were obtained from coconut and palm shells activated with NaOH.

Thus, for samples AC-OP, AC-ON, AC-CP, and AC-CN, average pore diameters of 19.4–20.2 Å were observed, consistent with their microporous nature. In contrast, the AC-OS sample showed an extremely low specific surface area (1.4 m^2^∙g^−1^), a small pore volume (0.005 cm^3^∙g^−1^), and a large average pore diameter (137.1 Å), indicating insufficient activation or partial structural collapse. These porosity characteristics directly influence the adsorption performance of the materials. Specifically, high surface area provides abundant active sites for adsorbate binding, while total pore volume determines the overall capacity. The distribution of micro- and mesopores affects both adsorption kinetics and accessibility: micropores favor strong adsorption of small molecules such as PCM, whereas mesopores facilitate diffusion and adsorption of larger molecules such as SMX. Furthermore, the mesoporous structure supports the integration of photocatalytic sites, enabling efficient contact between adsorbates and reactive centers, which is essential for the development of multifunctional materials combining adsorption and photocatalysis.

#### 2.1.2. Chemistry Surface-Properties of the Activated Carbons

The results of elemental analysis (Table S1) revealed a clear correlation between the synthesis conditions (precursor nature and activator type) and the chemical composition of the obtained carbons. For the AC-OP and AC-ON samples, the carbon content was 73.70–76.14% by mass, with the AC-ON sample having the highest nitrogen content (2.45% by mass), which is explained by the partial fixation of nitrogen-containing groups during acid activation. Similarly, for corn-based samples (AC-CP and AC-CN), the carbon content reached 76.08–77.49% by mass, with a nitrogen concentration in AC-CN of up to 1.93% by mass.

The elemental analysis data directly correlate with the measured zero charge point (pH_PZC_) values and the type of activator used (Figure S2). For materials activated with HNO_3_ and H_3_PO_4_ (AC-CN, AC-ON, AC-CP, AC-OP), the pH_PZC_ values are in the range of 5.2–6.4, indicating a moderately acidic surface. This is quantitatively confirmed by a higher content of heteroatoms (O and N) and the presence of specific functional groups. In contrast, alkaline activation (samples AC-OS and AC-CS) led to a significant decrease in the content of sulfur (to 0.03–0.06% by mass) and carbon (to 64.75% for AC-OS), which is consistent with the destruction of oxygen- and sulfur-containing groups in an alkaline environment [41,74,75,76]. As a result, the pH_PZC_ values for these samples shifted to the alkaline region (9.1–9.2), indicating the dominance of basic centers, such as pyron and phenolic structures, with a simultaneous decrease in the concentration of acidic oxygen- containing groups [77,78].

**Figure 2:**
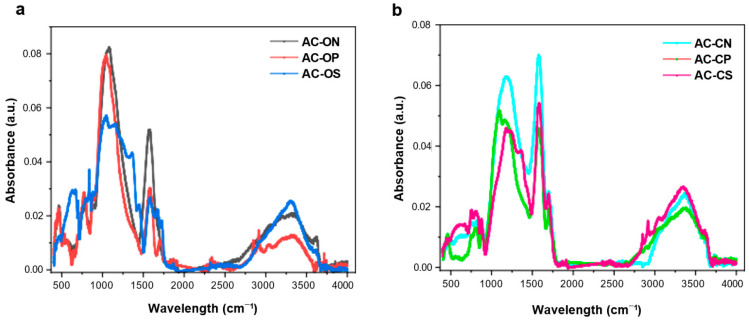
FT-IR spectra of the synthesized activated carbons prepared from (**a**) onion waste and (**b**) corn cob.

The presence of these functional groups was further confirmed by FTIR methods (Figure 2a,b). A broad peak in the 3400 cm^−1^ region in all spectra corresponds to valence vibrations of hydroxyl groups (-OH) or adsorbed water. A weak absorption band in the range 1700–1600 cm^−1^ is characteristic of C=O (carbonyl) and/or C=C (aromatic) vibrations [79]. The intense peak at 1200–1000 cm^−1^ in all cases refers to C-O vibrations in phenolic or ether groups, as well as to P-O bonds resulting from the activation of H_3_PO_4_. Finally, vibrations in the region below 1000 cm^−1^ are associated with deformation vibrations of aromatic rings. Thus, the combination of elemental analysis, pH_PZC_ and FTIR data allows us to conclude that the acid–base nature of the surface is strictly determined by the composition of the precursor and the chemical action of the activating agent.

The thermogravimetric analysis of all the synthesized activated carbons showed a high thermal stability at low temperatures. The weight loss values were 4.76, 8.64, and 14.74% for AC-OP, AC-ON, and AC-OS samples, respectively, and 14.70, 8.64, and 13.72% for AC-CP, AC-CN, and AC-CS samples. In both cases, for activated carbons where NaOH was used as activating agent, the weight loss was approximately of 14%. Moreover, when HNO3 was used in the chemical activation, the measured total weight loss was about 8%. Indeed, for all the samples, the weight loss was uniform and gradual, starting from 100 °C and gradually increasing up to 700 °C. The weight loss observed at this temperature range can be attributed to the removal of residual moisture or volatile compounds on the material’s structure after the thermal activation process [80].

In the same way, the TG profiles of the activated carbons after adsorption of SMX and PCM were obtained: after adsorption of SMX for AC-OP 6.1%, AC-ON 8.6%, and AC-OS 13.97%. According to the literature [81], it can be concluded that the weight loss of the materials after the adsorption process was similar to that obtained for the fresh carbon. This could be explained by the fact that SMX (according to Ola Svahn and Erland Björklund’s [82] decomposition rates of 133, 266 and 378 °C) forms dimers/polymers, i.e., by the process of dimerization upon heating and subsequent decomposition of the formed dimers. Thus, SMX formed dimers on the surface of the material, with the molecules bonding to each other when heated, which are difficult to remove from the carbon surface.

Regarding the TG analysis of the activated carbons after the adsorption of PCM, the following results were obtained: AC-OP 5%, AC-ON 8.8%, and AC-OS 13%. Weight loss values observed before and after adsorption of PCM were also similar, which allowed us to conclude that (referring to the studies of Nguyen DT et al. [83]) the adsorbed pollutant formed strong n-π, π-π-π, H-bonds with the surface of the material, which were not removed by increasing the temperature (decomposition temperature of 364 °C, according to Jendrzejewska et al., 2020) [84]. The results are shown in Figure S3 (Supplementary Materials).

#### 2.1.3. Morphological Properties of the Activated Carbons

The scanning electron microscopy (SEM) micrographs of the synthesized activated carbons can be seen in Figure S4a–f of the Supplementary Materials. SEM analysis revealed that the activated carbons obtained from onion waste (AC-OP, AC-ON, AC-OS) exhibited a homogeneous surface with layered roughness. On the other hand, carbon materials prepared from corn cobs (AC-CP, AC-CN, AC-CS) showed a denser structure. In this case, the layers adhered more tightly, the surface showed fine roughness, and the pores were evenly distributed. This indicated the formation of a more ordered pore framework, as has been previously reported in the literature [32,34].

### 2.2. PCM and SMX Adsorption Tests

#### 2.2.1. Kinetic Adsorption Studies

Adsorption kinetic experiments using single-pollutant (PCM, SMX) solutions were carried out. Then, the experimental data were fitted by using the kinetic models shown in Equations (1)–(3).

Pseudo-first order model:

(1) $$q_{t}=q_{e}\cdot \left(1-e^{-k_{1}t}\right)$$

Pseudo-second order model:

(2) $$\frac{t}{q_{t}}=\frac{1}{q_{e}^{2}k_{2}}+\frac{t}{q_{e}}$$

Elovich model:

(3) $$q_{t}=\frac{1}{\beta }\ln\left(\alpha \beta \right)+\frac{1}{\beta }\ln t$$

where *t* is the operation time (min), *q_t_* is the adsorption capacity at any time (mg/g), *q_e_* is the adsorption capacity at equilibrium time (mg/g), *k*_1_ is the rate constant for pseudo-first order model (min^−1^), *k*_2_ is the pseudo-second order model kinetic constant (g/(mg/min)), and α (mg/g min) and β (g/mg) are the initial rate of adsorption and the coefficient related to the activation energy in the Elovich model.

**Figure 3:**
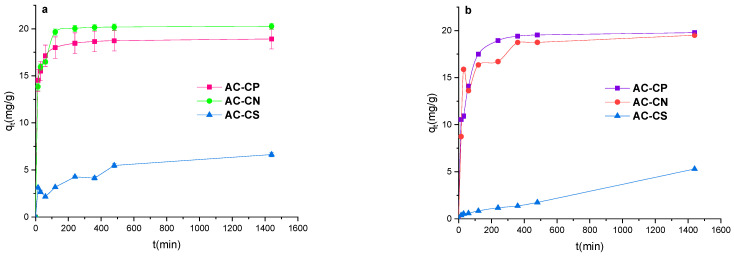
Adsorption kinetic curves of (**a**) PCM and (**b**) SMX onto the carbon materials synthesized from corn cob. Conditions: [Adsorbent dose] = 2.5 g∙L^−1^, C_0_ = 50 mg∙L^−1^, 200 rpm, pH = 6.3, T = 21 °C.

**Figure 4:**
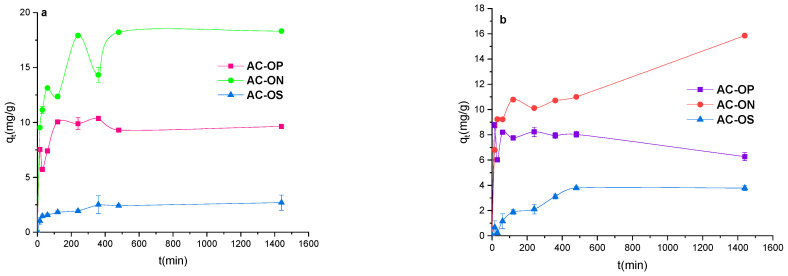
Adsorption kinetic curves of (**a**) PCM and (**b**) SMX onto the carbon materials synthesized from onion waste. Conditions: [Adsorbent dose] = 2.5 g∙L^−1^, C_0_ = 50 mg∙L^−1^, 200 rpm, pH = 6.3, T = 21 °C.

According to the experimental data obtained, the equilibrium time can be established in 8 h for all cases (see Figure 3a,b and Figure 4a,b). AC-CN activated carbon exhibited a good behavior for PCM adsorption, obtaining an adsorption capacity value of 20.3 mg/g. At the same time, the AC-OS and AC-CS samples showed significantly low values, e.g., 2.6 and 6.6 mg/g, respectively. These differences in the adsorptive performance are due to the different porous structures of the materials. In this sense, the AC-CN sample showed a high specific surface area (S_BET_ = 462 m^2^∙g^−1^) and a well-developed porous structure, with a high contribution of microporosity (V_micro_ = 0.182 cm^3^∙g^−1^), leading to an increase in the adsorptive removal of the pollutant in its inner porous structure. On the contrary, AC-OS material showed a specific surface area close to zero (1.4 m^2^∙g^−1^), indicative of the absence of porous structure, which explains its low ability for adsorption.

Similar findings were obtained for SMX adsorption, with adsorption capacity values of 19.8 and 19.5 mg∙g^−1^ for AC-CP and AC-CN samples, respectively. These materials showed high S_BET_ values (462 and 335 m^2^∙g^−1^, respectively), as well as relevant micropores contribution (V_micro_ = 0.182 and 0.129 cm^3^∙g^−1^, respectively). While the relationship between molecular sizes (8.0 × 5.0 × 3.5 Å for PCM, and 8.9 × 8.0 × 7.2 Å for SMX [41,85]) and pore size clearly controls access to the porous structure (steric hindrance effects), the surface chemistry of the carbon materials also plays a key role. For instance, AC-ON (S_BET_ = 397 m^2^∙g^−1^; V_micro_ = 0.158 cm^3^∙g^−1^) showed a high content of oxygenated functionalities (pH_PZC_ = 5.2), leading to a lower SMX adsorption capacity (*q*_exp_ = 11.0 mg∙g^−1^) compared to PCM (*q*_exp_ = 18.3 mg∙g^−1^). These results agree with DFT calculations reported in a later section. The calculated kinetic parameters for PCM and SMX adsorption are collected in Table 3 and Table 4.

To strengthen the validation of the kinetic models beyond the correlation coefficient (R^2^), a comprehensive error analysis was performed using the Sum of Squared Errors (SSE) and the Root Mean Square Error (RMSE). The fitting of the experimental data to the pseudo-second-order (PSO) model provided the best results for most systems. For example, for AC-CP and AC-CN carbons, the PSO model yielded the highest R^2^ values (0.9815 and 0.9505, respectively), which were consistently supported by significantly lower SSE and RMSE values compared to the pseudo-first-order and Elovich models. A similar trend has been found for the adsorption of pharmaceuticals onto waste-derived activated carbons [86,87]. In this study, the PSO model is interpreted not simply as a mathematical tool but as confirmation of the contribution of chemical interactions to the rate-limiting stage. This is consistent with the DFT results (Section 3.4), demonstrating specific interactions (e.g., π-π stacking and hydrogen bonds) characteristic of processes with a significant proportion of chemisorption.

#### 2.2.2. Competitive Kinetic Adsorption Studies

Binary adsorption studies of PCM-SMX solutions were accomplished. Equation (4) was used to calculate the equilibrium adsorption capacity (of each compound).

(4) $$q_{e,n}=\frac{\left(C_{0,n}-C_{e,n}\right)\cdot V}{m}$$

where *C*_0,*n*_ (mg/L) is the initial concentration of each compound (PCM or SMX), *C_e,n_* (mg/L) is the equilibrium concentration of each compound (PCM or SMX), *V* (L) is the volume of the solution, and *m* (g) is the weight of adsorbent. Equations (5) and (6) were used to calculate the total amount of adsorption capacity for the tested pollutants.

(5) $$q_{e,total}=\Sigma_{n=1}q_{e,n}$$

then,

(6) $$q_{e,total}=\frac{\left(C_{0,1}-C_{e,1}\right)\cdot V}{m}+\frac{\left(C_{0,2}-C_{e,2}\right)\cdot V}{m}$$

for both pollutants 1 and 2, in this case, PCM and SMX, respectively. Equation (7) was used to determine the change in adsorption capacity in binary systems compared to single systems.

(7) $$\Delta q_{e}(\%)=\frac{q_{e,\mathrm{single}}-q_{e,binary}}{q_{e,\mathrm{single}}}\cdot 100$$

where *q*_*e*,single_ is the equilibrium adsorption capacity in a system with one contaminant and *q*_*e*,binary_ is the equilibrium adsorption capacity in a binary system.

**Figure 5:**
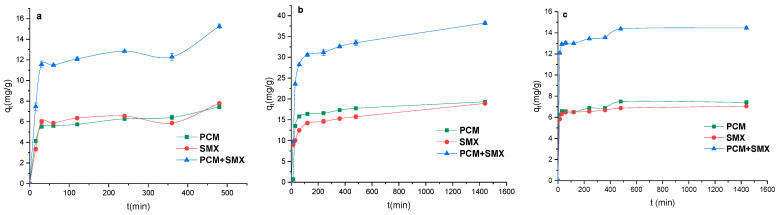
Adsorption kinetic curves of PCM-SMX onto the activated carbons synthesized from onion waste (**a**) AC-OP, (**b**) AC-ON, (**c**) AC-OS. Conditions: [Adsorbent dose] = 2.5 g∙L^−1^, C_0_ = 50 mg∙L^−1^ each compound, 200 rpm, pH = 6.3, T = 21 °C.

**Figure 6:**
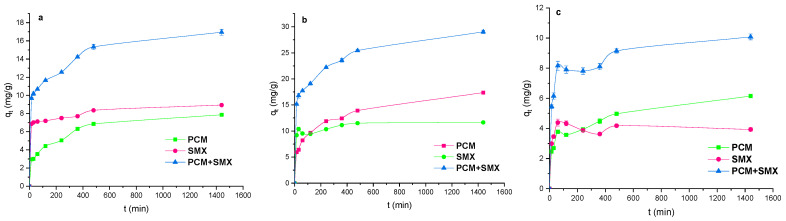
Adsorption kinetic curves of PCM-SMX onto the activated carbons synthesized from corncob (**a**) AC-CP, (**b**) AC-CN, (**c**) AC-CS. Conditions: [Adsorbent dose] = 2.5 g∙L^−1^, C_0_ = 50 mg∙L^−1^ each compound, 200 rpm, pH = 6.3, T = 21 °C.

The adsorption of PCM and SMX in binary systems demonstrates a complex interplay between competition and cooperative effects, revealing a transition in mechanisms depending on the carbon precursor (Figure 5a–c and Figure 6a–c). For AC-OP, AC- CP, and AC-CN samples, typical competitive behavior was observed (Δ*q_e_* > 0), where individual capacities decreased compared to single-component tests due to active site overlap and pore blocking. For instance, SMX capacity on AC-CP dropped significantly in the binary mixture. In contrast, for AC-ON and AC-OS materials, a significant increase in the uptake of both components was recorded (Δ*q_e_* < 0). While such high negative values—reaching −185.6% for PCM on AC-OS and −70.8% for SMX on AC-ON—might suggest a synergistic effect, we interpret this primarily as a cooperative adsorption phenomenon (Table 5) [88,89,90]. Nevertheless, the consistency of this trend across replicates, supported by the low SSE and RMSE values in the kinetic fitting (Table 6), suggests that the presence of a second component significantly alters the adsorption environment, potentially through enhanced π-π stacking or changes in the hydration shell [91,92,93,94]. The kinetic analysis of binary adsorption confirms that the pseudo-second-order (PSO) model remains the most accurate description for all studied carbons (R^2^ > 0.92; minimal RMSE values), which confirms the dominant role of chemical interactions as the rate-limiting step. Notably, for systems exhibiting cooperative effects (AC-ON, AC-OS), a marked acceleration of the adsorption kinetics was observed (higher *k*_2_ values), while competitive systems (AC-CP, AC-CN) showed a characteristic slowdown in the process dynamics.

#### 2.2.3. Isotherm Adsorption Studies

Several adsorption models have been used for the fitting of the experimental isotherm adsorption data. Among them, the most commonly used are the Freundlich and Langmuir equations. Also, the Dubinin–Radushkevich model (D-R) is usually used to describe the exothermic and endothermic nature of the adsorption processes, and the Guggenheim–Anderson–de Boer (GAB) equation is very useful in the prediction of multilayer adsorption isotherms [95].

The Freundlich model can be expressed by Equation (8):

(8) $$q_{e}=K_{F}\cdot C_{e}^{1/n_{F}}$$

where *K_F_* (L/g) and *n_F_* are Freundlich constants. The value of 1/*n_F_* is representative of the adsorption intensity or the adsorbent surface heterogeneity. Thus, the Langmuir model can be described by Equation (9):

(9) $$q_{e}=\frac{q_{\max}\cdot K_{L}\cdot C_{e}}{1+K_{L}\cdot C_{e}}$$

where *q*_max_ (mg/g) is the maximum adsorbate uptake and *K_L_* (L/mg) is a constant related to the affinity between adsorbate and adsorbent. Dubinin–Radushkevich (D-R) equation can be expressed as follows:

(10) $$q_{e}=q_{m}\cdot \exp\left(-\beta \cdot \varepsilon^{2}\right)$$

where *q_m_* (mg/g) is the theoretical adsorption capacity, and β (mol^2^/kJ^2^) is a constant associated with the adsorption energy. Also, the ε parameter refers to the Polanyi adsorption potential, which can be estimated by the following expression:

(11) $$\varepsilon =R\cdot T\cdot \ln\left(1+\frac{1}{C_{e}}\right)$$

In this sense, the calculated adsorption energy values can allow us to determine the type of adsorption process; thus, <8 kJ/mol usually refers to physical adsorption; 8–16 kJ/mol is usually associated with ion exchange; and a value higher than 16 kJ/mol indicates possible chemisorption.

Finally, Guggenheim–Anderson–de Boer (GAB) model is an extension of BET theory, mainly described for the accurate description of multilayer adsorption isotherms, not only in the gas phase, but also for liquid-phase systems. The GAB model can be described by the following equation:

(12) $$q_{e}=\frac{q_{m}\cdot K_{1}\cdot C_{e}}{\left(1-K_{2}\cdot C_{e}\right)\cdot \left[1+\left(K_{1}-K_{2}\right)\cdot C_{e}\right]}$$

where *q_m_* (mg/g) is the maximum adsorption capacity on the first monolayer and *K*_1_ and *K*_2_ (L/mg) are the equilibrium constants for the first and the second monolayer, respectively.

The standard Gibbs energy was calculated using the formula:

(13) $$\Delta G^{0}=\;-RTln(K_{c})$$

where *R* (mol·K) is universal gas constant, *T* (°C) is the operating temperature, and *K_c_* is the dimensionless equilibrium constant.

**Figure 7:**
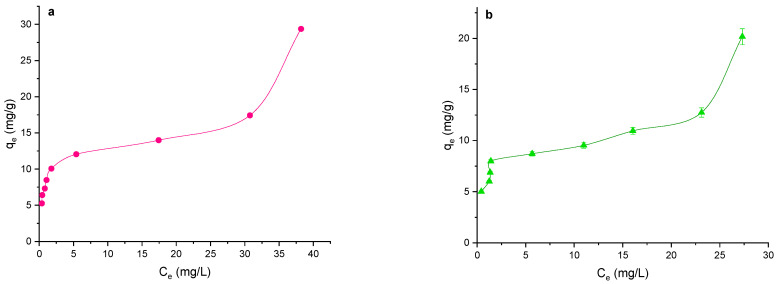
Adsorption isotherms of (**a**) PCM and (**b**) SMX onto AC-CN activated carbon. Conditions: [Adsorbent dose] = 0.115–1.0 g∙L^−1^, C_0_ = 50 mg∙L^−1^, 200 rpm, pH = 6.3, T = 21 °C.

**Figure 8:**
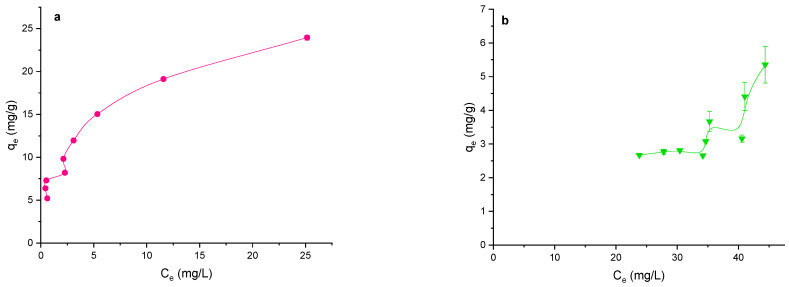
Adsorption isotherms of (**a**) PCM and (**b**) SMX onto AC-ON activated carbon. Conditions: [Adsorbent dose] = 0.115–1.0 g∙L^−1^, C_0_ = 50 mg∙L^−1^, 200 rpm, pH = 6.3, T = 21 °C.

The obtained experimental adsorption isotherms of PCM and SMX onto AC-CN and AC-ON activated carbons can be seen in Figure 7 and Figure 8, respectively.

For PCM adsorption onto AC-ON, an equilibrium adsorption capacity of 23.9 mg∙g^−1^ was determined; in this case, the best fit was found with the D-R model (*q_m_* = 24.71 mg∙g^−1^, R^2^ = 0.9371), while for SMX, the experimental adsorption capacity values obtained with AC-ON and AC-CN samples were 5.4 and 20.2 mg∙g^−1^, respectively. Likewise, the maximum equilibrium adsorption capacity was found for PCM adsorption onto AC-CN carbon (*q_e_* = 29.4 mg∙g^−1^); in this case, the GAB model described the experimental isotherm best (R^2^ = 0.9394), which is in agreement with the observed multilayer adsorption profile (Table 7). However, it is important to note that the classical Langmuir model was unable to accurately describe the adsorption in certain cases, resulting in non-physical mathematical parameters (e.g., theoretically infinite *q*_max_). This mathematical divergence is consistent with the visual S-shaped profile of the experimental isotherms (Type V according to IUPAC classification), indicating that the adsorbent surface has not reached full saturation (plateau) in the studied concentration range. Such behavior demonstrates that the adsorption is not limited to simple monolayer coverage but involves cooperative effects and complex multilayer formation. Therefore, for these specific systems, the maximum experimental uptake (*q*_exp_) is a more reliable indicator of the adsorbent’s capacity. Overall, it can be concluded that the D–R model is suitable for describing microporous filling and a dominant monolayer, while the GAB and Freundlich models more adequately characterize heterogeneous surfaces and cooperative multilayer adsorption processes [96].

As shown in Table 8, the negative Gibbs free energy (ΔG) values for most systems, ranging from −18.55 to −28.41 kJ·mol^−1^, indicate that adsorption is a spontaneous and energetically favorable process. The magnitude of these values suggests a physical adsorption mechanism enhanced by specific interactions, such as π-π stacking and hydrogen bonding, which is in excellent agreement with the DFT results. The positive ΔG value observed for PCM adsorption onto the AC-ON sample correlates with the extremely low sorption capacity obtained experimentally, suggesting that the process is energetically unfavorable under the studied conditions for this specific adsorbent.

### 2.3. Adsorption Tests Using an Environmentally Relevant Aqueous Matrix

Unlike model systems, real water matrices contain a complex mixture of organic and inorganic impurities that significantly affect adsorption efficiency. To confirm the practical applicability of the developed materials, the adsorption properties of the AC-CN sample were tested on wastewater from treatment plants (WWTPs). The physicochemical characteristics of the matrix (presented in Table S2) indicate a high initial concentration of total organic carbon (TOC)—1106 mg·L^−1^. Upon reaching equilibrium, a 16% decrease in TOC concentration was recorded.

Such a moderate decrease is explained by the complex influence of matrix effects. First, the presence of dissolved organic matter (DOM) and protein fractions creates fierce competition for the active centers of the adsorbent [97,98,99,100]. The mechanism of this influence is twofold: small DOM molecules directly compete with PCM and SMX for specific binding sites, while larger macromolecules cause blockage of micropores, limiting the access of target pollutants to the internal surface of the coal. Secondly, high salt concentrations in wastewater (ionic strength) can lead to the shielding of electrostatic interactions between the AC-CN surface and pharmaceutical molecules, which also reduces the overall capacity.

The result of a 16% reduction in TOC at an extremely high initial load (over 1000 mg/L) is consistent with the literature, where typical values for complex real-world effluents range from 10 to 25% when using comparable doses of adsorbents. The authors [97,98,99,100] emphasize that even with a significant influence of DOM and competitive ions, activated carbons from biomass retain their selectivity towards micropollutants, making them a promising tool for tertiary treatment of wastewater.

### 2.4. DFT Studies: Modeling of Adsorption Mechanism

**Scheme 1:**
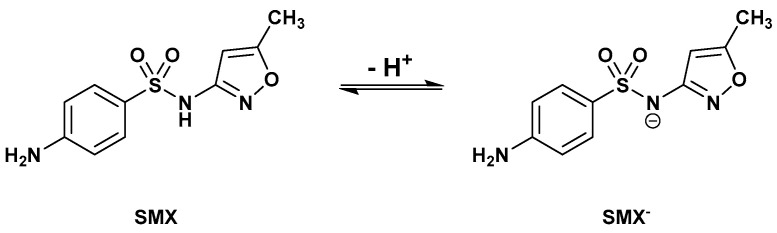
Dissociation of SMX in aqueous medium into SMX^−^ anion.

Geometry optimization calculations for PCM and SMX were performed to support the interpretation of the results. It should be noted that, under the working conditions (pH = 6.3), PCM remains neutral (pK_a_ = 9.38 [101]), while SMX is partially dissociated (pK_a_ = 5.7 [102]), as shown in Scheme 1.

The Henderson–Hasselbalch equation (Equation (14)) reveals that a significant fraction of SMX is present in its anionic form (Equation (15)).

(14) $$\log\left(\frac{\left[SMX^{-}\right]}{\left[SMX\right]}\right)=pH-pK_{a}=6.3-5.7=0.6\;\;\to \;\;\frac{\left[SMX^{-}\right]}{\left[SMX\right]}=10^{0.6}=3.981$$

(15) $$f_{SMX^{-}}=\frac{\left[SMX^{-}\right]}{\left[SMX^{-}\right]+\left[SMX\right]}=\frac{\frac{\left[SMX^{-}\right]}{\left[SMX\right]}}{1+\frac{\left[SMX^{-}\right]}{\left[SMX\right]}}=\frac{3.981}{4.981}\approx 0.80$$

Thus, 80% of the SMX molecules will be negatively charged. This will hinder SMX^−^ adsorption due to some electrostatic repulsion, since the pH at the point of zero charge (pH_pzc_) in the main samples (AC-ON and AC-CN) indicates that, at pH = 6.3, the adsorbent surface will exhibit an overall negative charge.

**Figure 9:**
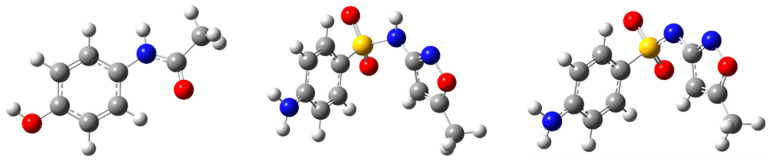
3D optimized structures of paracetamol (PCM, **left**), sulfamethoxazole (SMX, **center**) and sulfamethoxazole anion (SMX^−^, **right**). Atoms: C (gray), H (white), N (blue), O (red), S (yellow).

The minimum energy conformations are shown in Figure 9, with their cartesian coordinates presented in Tables S3–S5 of the Supplementary Materials. Tables S6 and S7 provide additional information (energies, electronic parameters, and chemical descriptors).

**Figure 10:**
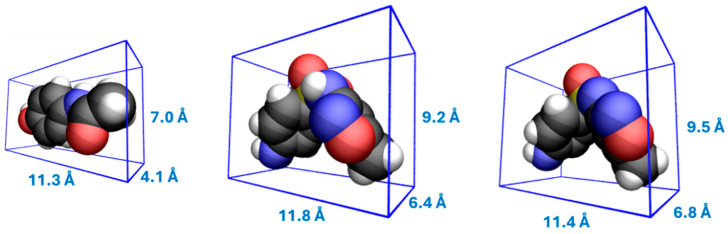
Van der Waals bounding boxes of PCM (**left**), SMX (**center**) and SMX^−^ (**right**). Atoms: C (gray), H (white), N (blue), O (red), S (yellow).

Then, the dimensions of the studied molecules were analyzed. Thus, Figure 10 shows how the SMX molecule (and its anion SMX^−^) has all dimensions larger than those of the PCM molecule. However, since the average pore diameter of both studied activated carbons is at least 19.4 Å, the species can easily enter the pores and reach the surface of the activated carbon.

**Figure 11:**
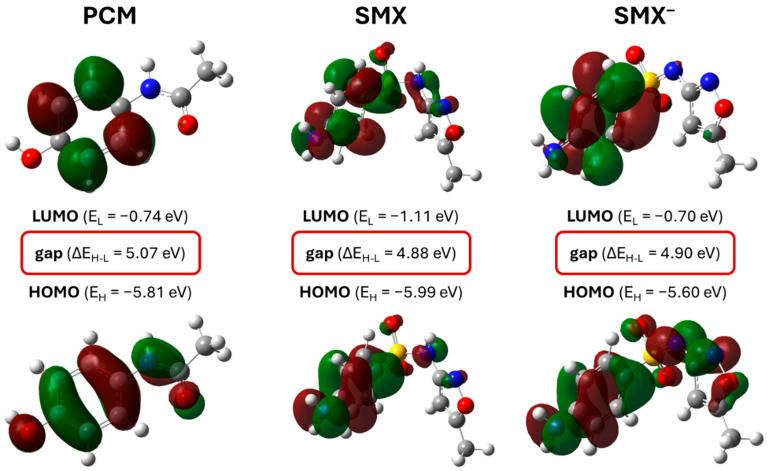
Plots of the HOMOs and LUMOs of PCM (**left**), SMX (**center**) and SMX^−^ (**right**). Atoms: C (gray), H (white), N (blue), O (red), S (yellow).

**Figure 12:**
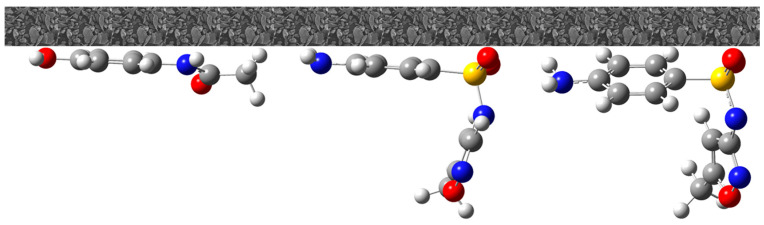
Possible interaction between PCM (**left**), SMX (**center**) and SMX^−^ (**right**) with the surface of the adsorbent material. Atoms: C (gray), H (white), N (blue), O (red), S (yellow).

The π-π and the n-π interactions involving the adsorbate’s π orbital would occur in the aromatic ring region, where the HOMOs and LUMOs are primarily located (Figure 11). PCM’s symmetric, flatter, more rigid structure allows both faces of its aromatic ring to engage in π-π stacking with activated carbon, additionally favoring multilayer formation, while SMX’s sulfonamide and isoxazole groups introduce steric hindrance on one face and greater conformational flexibility, potentially reducing interaction efficiency (see Figure 12). A shorter equilibrium adsorption distance, enabled by PCM’s planarity, likely enhances its adsorption stability, leading to higher adsorption energy than SMX. Furthermore, the presence of hydroxyl and carbonyl groups on activated carbon promotes the formation of hydrogen bonds, particularly in AC-ON and AC-CN samples, which exhibit higher FTIR spectral intensity in their corresponding signals (Figure 4) and superior adsorption results. The abundance of carbonyl groups suggests that the OH group of PCM, capable of forming stronger hydrogen bonds than the NH_2_ group of SMX [103], leads to more intense interactions with the activated carbon.

**Figure 13:**
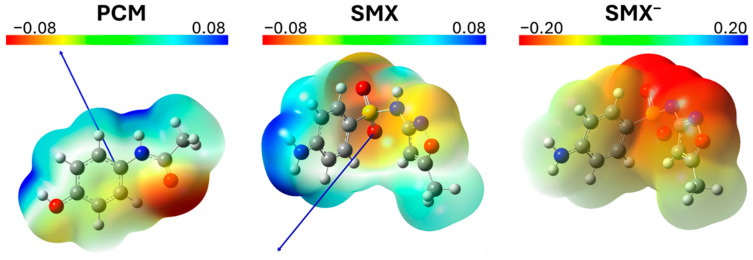
Molecular electrostatic potential (MEP, values in Hartree) of PCM, SMX and SMX^−^ and dipole moment vector of PCM and SMX. Atoms: C (gray), H (white), N (blue), O (red), S (yellow).

Another factor that could influence adsorption is the solvation of the adsorbates. The anionic form of SMX (SMX^−^), due to its delocalized negative charge, undergoes the most extensive solvation, which hinders desolvation and reduces its interaction efficiency with activated carbon. For neutral species, the polarity of an adsorbate can be characterized by the topological polar surface area (TPSA), which represents the sum of the surface areas of polar atoms in the molecule, mainly oxygen (O) and nitrogen (N), along with their associated hydrogen atoms capable of forming hydrogen bonds [104,105]. Paracetamol has a TPSA of 49.3 Å^2^, while for sulfamethoxazole, it is 107.0 Å^2^ [101]. This difference aligns with the DFT-calculated dipole moments, which are 7.7 D for PCM and 10.3 D for SMX (18.2 D for SMX^−^). As shown in Figure 13, molecular electrostatic potential (MEP) maps confirm that SMX displays broader regions of negative and positive potential than PCM, resulting in a stronger dipole moment. Consequently, neutral SMX experiences stronger aqueous solvation than PCM due to its higher TPSA and dipole moment, increasing the energy barrier for desolvation and limiting access to hydrophobic sites on activated carbon. These findings are consistent with the adsorption isotherms shown in Figure 7 and Figure 8, where SMX exhibited lower equilibrium adsorption capacities than PCM across all equilibrium concentrations. The reduced uptake of SMX can be attributed, at least in part, to its enhanced solvation, which raises the desolvation energy and restricts adsorption. In contrast, PCM, with lower polarity and weaker solvation, desolvates more readily, enhancing adsorption onto the carbon surface.

## 3. Materials and Methods

### 3.1. Materials

**Figure 14:**
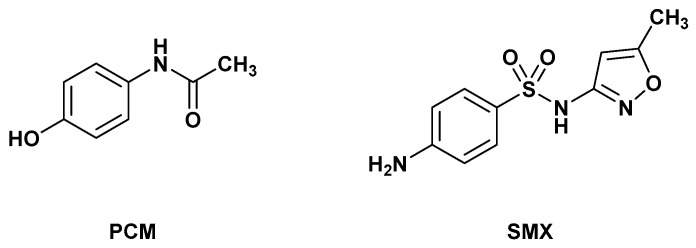
Molecular structures of PCM and SMX.

Sulfamethoxazole (SMX), with purity ≥99%; paracetamol (PCM), with purity ≥98%; and acetonitrile (HPLC grading, ≥99.9%) were purchased from Sigma-Aldrich (Merck KGaA, Darmstadt, Germany) and used without further purification. All chemicals were of analytical purity and were used directly in working solutions. The molecular structures of both studied molecules can be seen in Figure 14.

### 3.2. Preparation of the Activated Carbons

**Figure 15:**
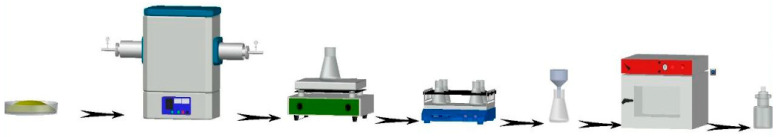
Scheme of the synthesis procedure of the activated carbons.

The synthesis of the activated carbons was adapted from previous work, as described elsewhere [36]. In this work, agro-industrial waste, such as onion waste and corn cobs, was used as raw material. To obtain activated carbons, the precursors were thoroughly washed with ultrapure water and then pre-carbonized at 450 °C in a vertical furnace under N_2_ flow (0.2 mL/min) for 1 h. Then, the material was pre-washed with a diluted HCl solution to remove residual impurities and activated by impregnation with acid solutions with a concentration of 3 M∙L^−1^ (HNO_3_, H_3_PO_4_) and alkali (NaOH). Finally, the obtained carbon materials were washed with ultrapure water until a neutral pH in the washing water was achieved. Then, the carbonization process was carried out at 600 °C under N_2_ flow (200 mL/min) for 1 h. The scheme of the synthesis procedure can be observed in Figure 15. The synthesized activated carbons were designated using the prefix AC-, followed by a two-letter suffix identifying the precursor and the activating agent, respectively. The first letter refers to the biomass source: O for onion peel and C for corn cob. The second letter indicates the chemical activating agent employed: P for H_3_PO_4_, N for HNO_3_, and S for NaOH. Accordingly, the six materials produced were labeled as AC-OP, AC-ON, AC-OS, AC-CP, AC-CN, and AC-CS.

### 3.3. Characterization Techniques

Textural properties of the carbon materials were obtained by N_2_ adsorption–desorption isotherms at −196 °C using a Micromeritics ASAP 2020 apparatus (Boynton Beach, FL, USA). The outgassing of the carbons was carried out at 150 °C for 12 h before each analysis. The specific surface area (S_BET_) was calculated by the Brunauer–Emmett–Teller (BET) equation; total pore volume (V_Total_) was estimated using the N_2_ volume adsorbed at P/P^0^ = 0.99; micropore volume (V_micro_) was determined by the *t-plot* method, and, finally, the average pore diameter (d_pore_) was measured at 4 V/A by BET.

Fourier-transformed infrared (FT-IR) spectra of the synthesized activated carbons were recorded using a Thermo Nicolet Nexus 670 spectrometer (Madison, WI, USA) in a wavelength range of 400–4000 cm^−1^, with a resolution of 2 cm^−1^. Moreover, the composition of the adsorbents was explored by elemental analysis using a LECO CHNS-932 elemental micro-analyser (LECO Corp., St. Joseph, MI, USA). Typically, 0.6–1.6 mg of sample was held in a furnace at 1000 °C, where combustion occurred. C, H, and N percentages (wt.%) were directly provided in the analysis, since O content was calculated by difference. The pH_PZC_ was determined by the pH drift method. To 50 mL of distilled water, 0.01 M NaCl and 0.01 M HCl were added to adjust the initial pH (pH_initial_) from 2 to 12. Then, 0.15 g of the sample was added, and the flasks were agitated in a shaker for 48 h. The final pH (pH_final_) was measured, and the pH_PZC_ was identified as the intersection point of the pH_final_ vs. pH_initial_ curve with the pH_final_ = pH_initial_ line [106]. The morphological properties of the materials were studied by the scanning electron microscopy (SEM) technique using a JEOL JSM 6400 microscope (JEOL Ltd., Akishima, Tokyo, Japan), equipped with a thermionic cathode and a 25 kV detector. Finally, the thermogravimetric analysis (TGA) of the carbon samples was performed using a thermogravimetric balance model LabSys Evo (Setaram Instrumentation, Lyon, France) under N_2_ atmosphere (50 mL/min), within a temperature range from 30–900 °C.

### 3.4. Adsorption Experiments

Batch adsorption experiments were performed in an orbital shaker under controlled and constant stirring (200 rpm) and room temperature. For the kinetic adsorption experiments, the following operation conditions were used: adsorbent dose of 2.5 g∙L^−1^, initial concentration of PCM or SMX of 50 mg∙L^−1^, solution volume of 100 mL, and placed in 250 mL Erlenmeyer flasks. In the same way, for the determination of the adsorption isotherms, an adsorbent dose ranging from 0.015 to 1.0 g∙L^−1^ was used, while the other conditions were the same as the kinetic tests. For batch adsorption experiments, the equilibrium adsorption capacity (*q_e_*) was calculated according to Equation (16):

(16) $$q_{e}=\frac{\left(C_{0}-C_{e}\right)\cdot V}{m}$$

where *q_e_* is the adsorption capacity at equilibrium time (mg∙g^−1^), C_0_ is the initial concentration of PCM or SMX (mg/L), C_e_ is the equilibrium concentration of PCM or SMX (mg∙L^−1^), V is the volume of solution (L), and m is the mass of activated carbon (g).

Samples were taken at different time intervals from 0 to 1440 min, until the equilibrium was reached. Then, the samples were centrifuged, filtered through 0.45 µm nylon membrane filters, and subsequently analyzed using a JASCO High-Performance Liquid Chromatograph (JASCO Corp., Tokyo, Japan) equipped with a UV-4575 detector (JASCO Corp., Tokyo, Japan). An Inertsil ODS-3 (GL Sciences Inc., Tokyo, Japan) (250 × 4.6 mm; 5 µm) column, and a mobile phase consisting of acetonitrile and ultrapure water (at pH 2.5) (45:55, *v*/*v*) were used. The flow rate was constant at 1.0 mL/min, and the detection was carried out at 254 nm.

To study the binary adsorption process, solutions with PCM and SMX (C_0_ = 50 mg∙L^−1^ each of them) were used. Additionally, samples of the real water matrices were collected from a WWTP effluent located in Taraz, Kazakhstan. TOC concentration of the real samples was measured by using a Shimadzu TOC-5000A analyser (Shimadzu Corp., Kyoto, Japan).

All adsorption experiments, including kinetic studies, equilibrium isotherms in single-component and binary systems, and thermodynamic tests, were performed in triplicate to ensure accuracy and reproducibility of data. Results are presented as mean values ± standard deviation (SD). Based on the kinetic screening results, only the two (AC-ON and AC-CN) best-performing carbons were selected for further detailed isotherm studies.

The experimental parameters for the adsorption studies were selected based on preliminary optimization tests and environmental relevance. The adsorbent dosage was fixed at 2.5 g∙L^−1^ as it provided a balance between sufficient removal efficiency and analytical precision. The initial pH of the solutions was maintained at its natural value (approximately 6.5–7.0) to simulate real-world water treatment conditions and to avoid the additional cost of pH adjustment, which is consistent with the sustainable approach of this work. The initial concentrations of PCM and SMX (50 mg∙L^−1^) were chosen to ensure accurate quantification while representing levels typically found in pharmaceutical-laden effluents.

### 3.5. DFT Studies: Methodology

DFT studies were conducted using the Gaussian 16 software [107], employing the MN12-SX functional [108] with the def2-TZVP basis set [109]. The SMD implicit solvation method [110] was applied to account for the effects of water as the solvent. The optimized structures and their cartesian coordinates were visualized using GaussView 6.0 [111], which was also used to illustrate the molecular electrostatic potential (MEP; at isovalue 0.02 a.u.), and at isovalue 0.03 a.u., the Highest Occupied Molecular Orbital (HOMO), and Lowest Unoccupied Molecular Orbital (LUMO). The dimensions of the molecular bounding box, based on Van der Waals radii, were calculated using Multiwfn 3.8 [112,113] and visualized with VMD 1.9 [114]. Information regarding the calculation of some DFT parameters can be found in the Supplementary Materials (Equations (S1)–(S8)).

## 4. Conclusions

Synthesized activated carbons obtained from agricultural waste (corn cobs and onion skins) demonstrated developed textural characteristics with a predominance of a microporous structure. A study of the adsorption of sulfamethoxazole (SMX) and paracetamol (PCM) from aqueous solutions showed that the best efficiency was demonstrated by the AC-CN (for PCM) and AC-CN/AC-CP (for SMX) samples. It was found that the adsorption capacity of the materials correlates with their specific surface area (S_BET_) = 335–462 m^2^·g^−1^) and the pronounced hydrophilic–hydrophobic nature of the surface. Kinetic equilibrium was achieved within a moderate time (~8 h), with the experimental data best described by a pseudo-second-order model. Two types of behavior were observed in binary systems: for a number of coals, a decrease in capacity was recorded due to competition for active centers, while in other cases, a synergistic effect was identified, caused by coadsorption and hydration interactions. The adsorption isotherms for AC-ON and AC-CN (with capacities ranging from 20.2 to 29.4 mg·g^−1^) are best described by the Dubinin–Radushkevich and GAB models. The exception was the SMX/AC-ON system (5.4 mg·g^−1^), where strong competition with water molecules was observed. Theoretical modeling using the DFT method confirmed the experimental data: the deprotonated state of SMX at natural pH (~80%) enhances its solvation, which hinders adsorption. In contrast, the flat structure of PCM promotes effective π–π stacking and the formation of hydrogen bonds with oxygen-containing groups of carbons.

Practical significance and prospects. The use of widely available agricultural waste (corn cobs and onion skins) as free precursors makes AC-ON and AC-CN production technology easily scalable and significantly reduces the overall cost compared to commercial fossil coal-based alternatives. The main operating costs are associated with chemical activation and energy consumption, but relatively low synthesis temperatures allow for optimization of energy costs. From a life cycle perspective, this technology minimizes environmental impact by converting agro-industrial waste into highly efficient adsorbents. Although the specific surface area of synthesized carbons is lower than that of some expensive commercial samples, their high selectivity towards pharmaceutical contaminants provides a competitive advantage. In addition, their high regeneration potential (e.g., through thermal desorption or solvent washing) makes these materials economically viable for long-term use in tertiary water treatment systems within the principles of the circular economy.

## Figures and Tables

**Table 2:** Textural properties of the synthesized activated carbons.

Material	S_BET_ (m^2^∙g^−1^)	V_Total_ (cm^3^∙g^−1^)	V_micro_ (cm^3^∙g^−1^)	V_meso_ (cm^3^∙g^−1^)	V_micro_/V_Total_	d_pore_ (Å)
AC-OP	289	0.146	0.113	0.033	0.77	20.2
AC-ON	397	0.198	0.158	0.040	0.79	19.9
AC-OS	1.4	0.005	0.001	0.004	0.20	137.1
AC-CP	335	0.163	0.129	0.034	0.79	19.5
AC-CN	462	0.224	0.182	0.042	0.81	19.4
AC-CS	251	0.125	0.094	0.031	0.75	20.0

**Table 3:** Kinetic parameters for SMX and PCM adsorption onto the activated carbons synthesized from onion waste.

Kinetic Model	SMX			PCM		
	AC-OP	AC-ON	AC-OS	AC-OP	AC-ON	AC-OS
*q_t_*^exp^ (mg∙g^−1^)	8.77 ± 0.9	11.02 ± 0.3	3.81 ± 0.1	9.63 ± 0.1	18.31 ± 0.04	2.69 ± 0.4
Pseudo-first-order						
*q_t_*^cal^ (mg∙g^−1^)	8.19	10.63	3.86	9.24	17.39	2.37
*K*_1_ (min^−1^)	0.097	0.140	0.005	0.035	0.036	0.028
R^2^	0.9923	0.9936	0.9610	0.9797	0.9482	0.9131
SSE	0.4565	0.6301	2.0801	1.6738	15.0732	0.5166
RMSE	0.2554	0.3000	0.5451	0.4890	1.4674	0.2717
Pseudo-second-order						
*q_t_*^cal^ (mg∙g^−1^)	8.40	10.78	4.53	9.82	18.57	2.59
*K*_2_ (g/(mg/min))	0.028	0.047	0.001	0.005	0.003	0.014
R^2^	0.9891	0.9917	0.9563	0.9935	0.9877	0.9663
SSE	0.6402	0.8208	2.4174	0.5345	3.5806	0.2005
RMSE	0.3024	0.3424	0.5877	0.2763	0.7152	0.1692
Elovich						
α (mg∙g^−1^min)	2.88 × 10^6^	1.51 × 10^14^	0.04	9.32	16.88	0.52
β (g∙mg^−1^)	2.64	3.73	0.94	0.88	0.46	2.66
R^2^	0.9779	0.9900	0.9344	0.9532	0.97191	0.9773
SSE	1.3003	0.9920	3.6378	0.8499	8.1726	0.1352
RMSE	0.4310	0.3764	0.7209	0.7416	1.0805	0.1390

**Table 4:** Kinetic parameters for SMX and PCM adsorption onto the activated carbons synthesized from corn cob.

Kinetic Model	SMX			PCM		
	AC-CP	AC-CN	AC-CS	AC-CP	AC-CN	AC-CS
*q_t_*^exp^ (mg∙g^−1^)	19.78 ± 0.1	19.49 ± 0.2	1.94 ± 0.3	18.91 ± 0.2	20.26 ± 0.05	6.63 ± 0.2
Pseudo-first-order						
*q_t_*^cal^ (mg∙g^−1^)	18.90	17.67	1.87	18.18	19.49	5.17
*K*_1_ (min^−1^)	0.03	0.05	0.004	0.093	0.069	0.012
R^2^	0.9470	0.9300	0.9310	0.9818	0.9651	0.6397
SSE	18.2931	23.0483	0.2348	5.2810	11.941	3.6552
RMSE	1.6166	1.8145	0.1832	0.8686	1.3061	0.7226
Pseudo-second-order						
*q_t_*^cal^ (mg∙g^−1^)	20.14	18.89	2.13	18.81	20.40	5.70
*K*_2_ (g/(mg min))	0.002	0.003	0.002	0.011	0.006	0.003
R^2^	0.9815	0.9505	0.9531	0.9977	0.9911	0.7499
SSE	6.3790	15.5812	0.1598	0.6668	3.0483	6.4670
RMSE	0.9546	1.4919	0.1511	0.3086	0.6600	0.9612
Elovich						
α (mg/g min)	14.24	37.53	0.03	2.43 × 10^5^	2.0 × 10^3^	0.44
β (g∙mg^−1^)	0.41	0.49	2.19	0.99	0.66	1.09
R^2^	0.9634	0.9368	0.9646	0.9915	0.9784	0.8771
SSE	12.6154	19.9009	0.1205	2.4575	7.3964	7.4370
RMSE	1.3425	1.6861	0.1312	0.5925	1.0280	1.0307

**Table 5:** Competitive and synergistic effects in PCM-SMX adsorption onto the synthesized activated carbons: single vs. binary systems.

Material	PCM			SMX		
	*q_e_*_,single_	*q_e_*_,binary_	Δ*q_e_* (%)	*q_e_*_,single_	*q_e_*_,binary_	Δ*q_e_* (%)
AC-OP	9.63	7.44	22.74	8.77	7.70	12.20
AC-ON	18.31	19.14	−4.53 *	11.02	18.83	−70.87 *
AC-OS	2.58	7.37	−185.66 *	3.81	6.99	−83.46 *
AC-CP	19.81	7.78	60.72	19.78	8.83	55.36
AC-CN	20.26	17.17	15.25	19.49	11.59	40.53
AC-CS	6.63	6.06	8.60	1.94	3.80	−95.88 *

* Negative Δ*q_e_* values indicate an increase in adsorption capacity in the binary system compared to the single system (synergistic effect).

**Table 6:** Kinetic parameters for the adsorption of PCM and SMX in binary systems onto the synthesized activated carbons.

Kinetic Model	SMX-PCM			SMX-PCM		
	AC-OP	AC-ON	AC-OS	AC-CP	AC-CN	AC-CS
*q_t_*^exp^ (mg∙g^−1^)	13.34 ± 0.2	37.97 ± 0.3	14.36 ± 0.07	16.61 ± 0.1	28.77 ± 0.2	9.85 ± 0.2
Pseudo-first-order						
*q_t_*^cal^ (mg∙g^−1^)	12.91	33.31	13.52	13.53	23.14	8.35
*K*_1_ (min^−1^)	0.06	0.03	0.14	0.06	0.05	0.05
R^2^	0.9857	0.9593	0.9861	0.8500	0.8463	0.9271
SSE	2.1271	95.0388	2.2383	27.6945	84.6663	4.8918
RMSE	0.5954	3.6847	0.5655	1.9891	3.4778	0.8360
Pseudo-second-order						
*q_t_*^cal^ (mg∙g^−1^)	13.27	36.19	13.82	14.55	25.08	8.83
*K*_2_ (g/(mg min))	0.01	0.001	0.03	0.006	0.003	0.01
R^2^	0.9292	0.9620	0.9925	0.9197	0.9223	0.9523
SSE	10.5404	46.8451	1.2151	14.8238	42.8015	3.2029
RMSE	1.3254	2.5869	0.4166	1.4552	2.4727	0.6764
Elovich						
α (mg/g min)	52.99	8.91	2.25 × 10^9^	26.01	17.75	31.92
β (g∙mg^−1^)	0.70	0.20	2.05	0.62	0.32	1.12
R^2^	0.9561	0.9228	0.9974	0.9866	0.9905	0.9684
SSE	6.5303	50.1278	0.4248	2.4716	5.2620	2.1240
RMSE	1.0432	2.6760	0.2463	0.5942	0.8670	0.5508

**Table 7:** Adsorption isotherm parameters for SMX and PCM adsorption onto AC-ON and AC-CN activated carbons.

Isotherm Model	SMX		PCM	
	AC-ON	AC-CN	AC-ON	AC-CN
*q*_exp_ (mg∙g^−1^)	5.4 ± 0.2	20.2 ± 0.3	23.9 ± 0.04	29.4 ± 0.01
Langmuir				
*q*_max_ (mg∙g^−1^)	–	246.54	–	14.39
*K_L_* (L∙mg^−1^)	0.03	0.007	1.6 × 10^−6^	0.63
R^2^	0.9911	0.8562	0.9128	0.5854
Freundlich				
*K_F_* (L∙g^−1^)	0.04	4.20	0.82	5.65
*n_F_*	0.81	1.49	0.94	3.29
R^2^	0.6433	0.8867	0.9154	0.7604
D-R				
*q_m_* (mg∙g^−1^)	5.81	64.99	24.71	12.37
β (mol^2^/kJ^2^)	626.33	286.02	89.90	1.47
R^2^	0.5127	0.7409	0.9371	0.4242
E_D-R_ (kJ/mol)	8.01	8.90	14.01	15.81
GAB				
*q_m_* (mg∙g^−1^)	3.65	7.00	-	-
*K*_1_ (mg∙L^−1^)	0.02	8.32	1.65 × 10^−4^	3.71 × 10^−4^
*K*_2_ (mg∙L^−1^)	0.4 × 10^−3^	0.02	0.01	0.01
R^2^	0.8205	0.9715	0.9310	0.9394

**Table 8:** Thermodynamic parameters (ΔG^0^) for the adsorption of PCM and SMX onto the synthesized activated carbons.

Adsorbent	Pollutant	*K_L_* (L·mg^−1^)	ΔG^0^ (kJ·mol^−1^)
AC-CN	PCM	0.63	−28.41
AC-CN	SMX	0.007	−18.55
AC-ON	PCM	1.6 × 10^−6^	+3.52
AC-ON	SMX	0.03	−22.14

## Data Availability

All data supporting the findings of this study are included within the article.

## Supplementary Materials

The following supporting information can be downloaded at https://www.mdpi.com/article/10.3390/molecules31071162/s1, DFT calculation parameters (S1–S8); Figure S1: Pores size distributions of the activated carbons obtained from (a) onion waste (b) corn cob; Figure S2. pH_PZC_ values of the activated carbons obtained from (a) onion waste (b) corn cob; Figure S3: TG profiles of the activated carbons prepared from (a) onion wastes (AC-OP) and after adsorption PCM, SMX, (b) onion wastes (AC-ON) and after adsorption PCM, SMX, (c) onion wastes (AC-OS) and after adsorption PCM, SMX, (d) corncob wastes (AC-CP, AC-CN, AC-CS); Figure S4: SEM micrographs of the synthesized activated carbons; Table S1: Elemental analysis of the synthesized activated carbons; Table S2: Physico-chemical parameters of the WWTP effluent tested; Table S3: Cartesian coordinates for optimized structure of paracetamol (MN12-SX/def2-TZVP, SMD solvation: water); Table S4: Cartesian coordinates for optimized structure of sulfamethoxazole (MN12-SX/def2-TZVP, SMD solvation: water); Table S5: Cartesian coordinates for optimized structure of sulfamethoxazole anion (MN12-SX/def2-TZVP, SMD solvation: water); Table S6: Energetic data for optimized structures of paracetamol (PCM), sulfamethoxazole (SMX) and sulfamethoxazole anion (SMX^−^) at MN12-SX/def2-TZVP level, with SMD solvation model; solvent: water; Table S7: Electronic parameters and chemical descriptors of PCM, SMX and SMX^−^ calculated at MN12- SX/def2-TZVP level considering water solvation (SMD method). Values in eV, except for S (eV^−1^).

## Abbreviations

The following abbreviations are used in this manuscript:

PCM	Paracetamol
SMX	Sulfamethoxazole
GAB	Guggenheim–Anderson–de Boer model
DFT	Density Functional Theory
D-R	Dubinin–Radushkevich model
CECs	Contaminants of emerging concern
ACs	Activated carbons
SEM	Scanning Electron Microscopy
BET	Brunauer–Emmett–Teller

## References

[1] Di Marcantonio C., Chiavola A., Gioia V., Leoni S., Cecchini G., Frugis A., Ceci C., Spizzirri M., Boni M.R. (2023). A step forward on site-specific environmental risk assessment and insight into the main influencing factors of CECs removal from wastewater. J. Environ. Manag..

[2] Rizzo L., Malato S., Antakyali D., Beretsou V.G., Đolić M.B., Gernjak W., Heath E., Ivancev-Tumbas P., Karaolia A.R.L., Ribeiro G. (2019). Consolidated vs. new advanced treatment methods for the removal of contaminants of emerging concern from urban wastewater. Sci. Total Environ..

[3] Gogoi A., Mazumder P., Tyagi V.K., Tushara Chaminda G.G., An A.K., Kumar M. (2018). Occurrence and fate of emerging contaminants in water environment: A review. Groundw. Sustain. Dev..

[4] Kim S., Chu K.H., Al-Hamadani Y.A.J., Park C.M., Jang M., Kim D.-H., Yu M., Heo J., Yoon Y. (2018). Removal of contaminants of emerging concern by membranes in water and wastewater: A review. Chem. Eng. J..

[5] Krzeminski P., Tomei M.C., Karaolia P., Langenhoff A., Almeida C.M.R., Felis E., Gritten F., Andersen H.R., Fernandes T., Manaia C.M. (2019). Performance of secondary wastewater treatment methods for the removal of contaminants of emerging concern implicated in crop uptake and antibiotic resistance spread: A review. Sci. Total Environ..

[6] Tiedeken E.J., Tahar A., McHugh B., Rowan N.J. (2017). Monitoring, sources, receptors, and control measures for three European Union watch list substances of emerging concern in receiving waters—A 20 year systematic review. Sci. Total Environ..

[7] Kaczala F., Blum S.E. (2016). The Occurrence of Veterinary Pharmaceuticals in the Environment: A Review. Curr. Anal. Chem..

[8] Richardson S.D., Ternes T.A. (2018). Water Analysis: Emerging Contaminants and Current Issues. Anal. Chem..

[9] Khan J.A., Sayed M., Khan S., Shah N.S., Dionysiou D.D., Boczkaj G. (2020). Chapter 9—Advanced oxidation processes for the treatment of contaminants of emerging concern, Contaminants of Emerging Concern in Water and Wastewater. Adv. Treat. Process..

[10] Clarke B.O., Smith S.R. (2011). Review of ‘emerging’ organic contaminants in biosolids and assessment of international research priorities for the agricultural use of biosolids. Environ. Int..

[11] Stackelberg P., Furlong E., Meyer M., Zaugg S., Henderson A., Reissman D. (2004). Persistence of pharmaceutical compounds and other organic wastewater contaminants in a conventional drinking-water-treatment plant. Sci. Total Environ..

[12] Chander V., Sharma B., Negi V., Aswal R., Singh P., Singh R., Dobhal R. (2016). Pharmaceutical Compounds in Drinking Water. J. Xenobiotics.

[13] Focazio M., Kolpin D., Barnes K., Furlong E., Meyer M., Zaugg S., Barber L., Thurman M. (2008). A national reconnaissance for pharmaceuticals and other organic wastewater contaminants in the United States--II) untreated drinking water sources. Sci. Total Environ..

[14] Sackey L., Okobeng A., Obidieh P., Ngala F., Otoo E., Quartey J., Bentil J., Azanu D. (2024). Risk Assessment of Pharmaceutical Contaminants in Pharmaceutical Wastewater. Sci. World J..

[15] Kalmakhanova M.S., Kurtebayeva A.A., Tleuova Z.T., Satybaldiev B., Orynbayev S.A., Malakar A., Gomes H.T., Snow D.D. (2025). Environmental Impact of Wastewater on Surface and Groundwater in Central Asia. Sustainability.

[16] Munzhelele E., Mudzielwana R., Ayinde W., Gitari W. (2024). Pharmaceutical Contaminants in Wastewater and Receiving Water Bodies of South Africa: A Review of Sources, Pathways, Occurrence, Effects, and Geographical Distribution. Water.

[17] De Oliveira M., Frihling B., Velasques J., Filho F., Cavalheri P., Migliolo L. (2019). Pharmaceuticals residues and xenobiotics contaminants: Occurrence, analytical techniques and sustainable alternatives for wastewater treatment. Sci. Total Environ..

[18] Couto C., Lange L., Amaral M. (2019). Occurrence, fate and removal of pharmaceutically active compounds (PhACs) in water and wastewater treatment plants—A review. J. Water Process Eng..

[19] Kolpin D.W., Furlong E.T., Meyer M.T., Thurman E.M., Zaugg S.D., Barber L.B., Buxton H.T. (2002). Pharmaceuticals, hormones, and other organic wastewater contaminants in U.S. streams, 1999-2000: A national reconnaissance. Environ. Sci. Technol..

[20] Ghazal H., Koumaki E., Hoslett J., Malamis S., Katsou E., Barceló D., Jouhara H. (2022). Insights into current physical, chemical and hybrid technologies used for the treatment of wastewater contaminated with pharmaceuticals. J. Clean. Prod..

[21] Kanakaraju D., Glass B., Oelgemöller M. (2018). Advanced oxidation process-mediated removal of pharmaceuticals from water: A review. J. Environ. Manag..

[22] Wong S., Lim Y., Ngadi N., Mat R., Hassan O., Inuwa I.M., Mohamed N.B., Low J.H. (2018). Removal of acetaminophen by activated carbon synthesized from spent tea leaves: Equilibrium, kinetics and thermodynamics studies. Powder Technol..

[23] Bizi M. (2020). Sulfamethoxazole Removal from Drinking Water by Activated Carbon: Kinetics and Diffusion Process. Molecules.

[24] Gutiérrez-Sánchez P., Navarro P., Alvarez-Torrellas S., García J., Larriba M. (2022). Extraction of neonicotinoid pesticides from aquatic environmental matrices with sustainable terpenoids and eutectic solvents. Sep. Purif. Technol..

[25] Haciosmanoğlu G.G., Mejías C., Martín J., Santos J.L., Aparicio I., Alonso E. (2022). Antibiotic adsorption by natural and modified clay minerals as designer adsorbents for wastewater treatment: A comprehensive review. J. Environ. Manag..

[26] Silva S., Kalmakhanova M.S., Massalimova B.K., Sgorlon J.G., de Tuesta J.L.D., Gomes H.T. (2019). Wet Peroxide Oxidation of Paracetamol Using Acid Activated and Fe/Co-Pillared Clay Catalysts Prepared from Natural Clays. Catalysts.

[27] Kumari S., Chowdhry J., Kumar M., Garg M.C. (2024). Zeolites in wastewater treatment: A comprehensive review on scientometric analysis, adsorption mechanisms, and future prospects. Environ. Res..

[28] Shimabuku K.K., Kearns J.P., Martinez J.E., Mahoney R.B., Moreno-Vasquez L., Summers R.S. (2016). Biochar sorbents for sulfamethoxazole removal from surface water, stormwater, and wastewater effluent. Water Res..

[29] García-Galán M.D.M., Fernández-Blanco C.A., Cuerda-Correa E.M., Garrido-Zoido J.M., Alexandre-Franco M.F. (2025). Waste Surgical Masks as Precursors of Activated Carbon: A Circular Economy Approach to Mitigate the Impact of Microplastics and Emerging Dye Contaminants. Materials.

[30] Calisto V., Jaria G., Silva C.P., Ferreira C.I.A., Otero M., Esteves V.I. (2017). Single and multi-component adsorption of psychiatric pharmaceuticals onto alternative and commercial carbons. J. Environ. Manag..

[31] Ndagijimana P., Liu X., Yu G., Wang Y. (2019). Synthesis of a novel core-shell-structure activated carbon material and its application in sulfamethoxazole adsorption. J. Hazard. Mater..

[32] Álvarez-Torrellas S., Peres J.A., Gil-Álvarez V., Ovejero G., García J. (2017). Effective adsorption of non-biodegradable pharmaceuticals from hospital wastewater with different carbon materials. Chem. Eng. J..

[33] Çaliskan E., Göktürk S. (2010). Adsorption Characteristics of Sulfamethoxazole and Metronidazole on Activated Carbon. Sep. Sci. Technol..

[34] Kheddo A., Rhyman L., Elzagheid M.I., Jeetah P., Ramasami P. (2020). Adsorption of synthetic dyed wastewater using activated carbon from rice husk. SN Appl. Sci..

[35] Bibi F., Hussain R., Iqbal N., Saeed S., Waseem M., Elkaeed E.B., Al-Anazy M.M., Haq S. (2023). Chemical activation and magnetization of onion waste derived carbon for arsenic removal. Arab. J. Chem..

[36] Torrellas S.Á., Lovera R.G., Escalona N., Sepúlveda C., Sotelo J.L., García J. (2015). Chemical-activated carbons from peach stones for the adsorption of emerging contaminants in aqueous solutions. Chem. Eng. J..

[37] Tonucci M.C., Gurgel L.V.A., de Aquino S.F. (2015). Activated carbons from agricultural byproducts (pine tree and coconut shell), coal, and carbon nanotubes as adsorbents for removal of sulfamethoxazole from spiked aqueous solutions: Kinetic and thermodynamic studies. Ind. Crop. Prod..

[38] Portillo E., Flores S., Carrizosa R., Álvarez-Torrellas S., Carbajo J., Águeda V.I., García J. (2025). Sustainable adsorbents from sewage sludge: Efficient removal of cytostatic compounds in single and complex aqueous matrices. J. Environ. Chem. Eng..

[39] Zhou L., Yu Q., Cui Y., Xie F., Li W., Li Y., Chen M. (2017). Adsorption properties of activated carbon from reed with a high adsorption capacity. Ecol. Eng..

[40] Kim D., Kil H., Nakabayashi K., Yoon S., Miyawaki J. (2017). Structural elucidation of physical and chemical activation mechanisms based on the microdomain structure model. Carbon.

[41] Heidarinejad Z., Dehghani M., Heidari M., Javedan G., Ali I., Sillanpää M. (2020). Methods for preparation and activation of activated carbon: A review. Environ. Chem. Lett..

[42] Nazem M., Zare M., Shirazian S. (2020). Preparation and optimization of activated nano-carbon production using physical activation by water steam from agricultural wastes. RSC Adv..

[43] Tobi A.R., Dennis J.O., Zaid H.M., Adekoya A.A., Yar A., Fahad U. (2019). Comparative analysis of physiochemical properties of physically activated carbon from palm bio-waste. J. Mater. Res. Technol..

[44] Hayashi J., Kazehaya A., Muroyama K., Watkinson A. (2000). Preparation of activated carbon from lignin by chemical activation. Carbon.

[45] Ruiz-Fernández M., Alexandre-Franco M., Fernández-González C., Gómez-Serrano V. (2011). Development of activated carbon from vine shoots by physical and chemical activation methods. Some insight into activation mechanisms. Adsorption.

[46] Macia-Agullo J., Moore B., Cazorla-Amorós D., Linares-Solano Á. (2004). Activation of coal tar pitch carbon fibres: Physical activation vs. chemical activation. Carbon.

[47] Bedia J., Peñas-Garzón M., Gómez-Avilés A., Rodriguez J., Belver C. (2020). Review on Activated Carbons by Chemical Activation with FeCl_3_. C.

[48] Gao Y., Yue Q., Gao B., Li A. (2020). Insight into activated carbon from different kinds of chemical activating agents: A review. Sci. Total Environ..

[49] Olivares-Marín M., Fernández-González C., Macías-García A., Gómez-Serrano V. (2012). Preparation of activated carbon from cherry stones by physical activation in air. Influence of the chemical carbonisation with H_2_SO_4_. J. Anal. Appl. Pyrol..

[50] Neme I., Gonfa G., Masi C. (2022). Activated carbon from biomass precursors using phosphoric acid: A review. Heliyon.

[51] Luo Y. (2018). The performance of phosphoric acid in the preparation of activated carbon-containing phosphorus species from rice husk residue. J. Mater. Sci..

[52] Higai D., Huang Z., Qian E.W. (2020). Preparation and surface characteristics of phosphoric acid-activated carbon from coconut shell. Environ. Prog. Sustain. Energy.

[53] Debbache H. (2024). A comprehensive analysis of the use of chemical activation technology to produce activated carbon from agricultural residues. Cellul. Chem. Technol..

[54] Hashem H. (2024). Inclusive study of peanut shells derived activated carbon as an adsorbent for removal of lead and methylene blue from water. Sci. Rep..

[55] Giraldo L., Vargas D.P., Moreno-Piraján J.C. (2020). Study of CO_2_ Adsorption on Chemically Modified Activated Carbon with Nitric Acid. Front. Chem..

[56] Ternero-Hidalgo J., Rosas J.M., Palomo J., Valero-Romero M.J., Rodríguez-Mirasol J., Cordero T. (2016). Functionalization of activated carbons by HNO_3_ treatment. Carbon.

[57] Wilkinson J., Boxall A.B.A., Kolpin D.W., Leung K.M.Y., Lai R.W.S., Galbán-Malagón C., Adell A.D., Mondon J., Metian M., Marchant R.A. (2022). Pharmaceutical pollution of the world’s rivers. Proc. Natl. Acad. Sci. USA.

[58] Hoang A., Do M.C., Kim K.-W. (2024). Environmental risk assessment of selected pharmaceuticals in hospital wastewater. Chemosphere.

[59] Hossen M., Sattar G.S., Mostafa M.G. (2024). Factors affecting the performance of a pharmaceutical wastewater treatment plant. Heliyon.

[60] Rosli F., Ahmad H., Jumbri K., Abdullah A.H., Kamaruzaman S., Abdullah N.A.F. (2021). Efficient removal of pharmaceuticals from water using graphene nanoplatelets as adsorbent. R. Soc. Open Sci..

[61] Madhusudhan A., Zelenka T., Satrapinskyy L., Roch T., Gregor M., Cheng P., Monfort O. (2025). A comparative study of different activation methods for hydrochar. Environ. Sci. Pollut. Res. Int..

[62] Zhang J., Ding J., Liu L.-M., Wu R., Ding L., Jiang J.-Q., Pang J.-W., Li Y., Ren N.-Q., Yang S.-S. (2023). Selective removal of sulfamethoxazole by a novel double Z-scheme photocatalyst. Environ. Sci. Ecotechnology.

[63] Upadhyay S., Rani N., Kumar V., Mythili R., Jain D. (2023). A review on simultaneous heavy metal removal and organo-contaminants degradation. Microbiol. Res..

[64] Briffa J., Sinagra E., Blundell R. (2020). Heavy metal pollution in the environment and their toxicological effects on humans. Heliyon.

[65] Madesh S., Sudhakaran G., Meenatchi R., Guru A., Arockiaraj J. (2024). Interconnected environmental challenges: Heavy metal–drug interactions. Drug Chem. Toxicol..

[66] Sultana E., Mamun M. (2025). Golden eyes on pollutants: Colorimetric detection of emerging contaminants with AuNPs. RSC Adv..

[67] Abu-Daabes M., Zeitoun A., Mazi W. (2023). Competitive Adsorption of Quaternary Metal Ions, Ni^2+^, Mn^2+^, Cr^6+^, and Cd^2+^, on Acid-Treated Activated Carbon. Water.

[68] Eskandari P., Farhadian M., Nazar A., Goshadrou A. (2020). Cyanide adsorption on activated carbon impregnated with ZnO, Fe2O3, TiO2 nanometal oxides: A comparative study. Int. J. Environ. Sci. Technol..

[69] Rathi T., Gomase V., Thawkar S., Ganvir P., Saravanan D., Jugade R. (2025). Novel ACS Film for Efficient Dual Adsorption of Cr(VI) and SDS: Mechanistic In-sights and Practical Applications. Environ. Res..

[70] Mergbi M., Aboagye D., Contreras S., Amor H., Medina F., Djellabi R. (2023). Fast g-C3N4 sonocoated activated carbon for enhanced solar photocatalytic oxidation of organic pollutants through Adsorb & Shuttle process. Ultrason. Sonochemistry.

[71] Bao Z., Lotfy V., Zhou X., Fu S., Basta A. (2024). Assessment of porous carbon from rice straw residues with potassium ferrate-assisted activation as cationic and anionic dye adsorbents. Ind. Crop. Prod..

[72] Blachnio M., Derylo-Marczewska A., Charmas B., Zienkiewicz-Strzalka M., Bogatyrov V., Galaburda M. (2020). Activated carbon from agricultural wastes for adsorption of organic pollutants. Molecules.

[73] Hafizuddin M.S., Lee C.L., Chin K.L., H’ng P.S., Khoo P.S., Rashid U. (2021). Fabrication of Highly Microporous Structure Activated Carbon via Surface Modification with Sodium Hydroxide. Polymers.

[74] Moreno-Castilla C. (2004). Adsorption of organic molecules from aqueous solutions on carbon materials. Carbon.

[75] Zhou F., Li K., Hang F., Zhang Z., Chen P., Wei L., Xie C. (2022). Efficient removal of methylene blue by activated hydrochar prepared by hydrothermal carbonization and NaOH activation of sugarcane bagasse and phosphoric acid. RSC Adv..

[76] Djilani C., Zaghdoudi R., Modarressi A., Rogalski M., Djazi F., Lallam A. (2012). Elimination of organic micropollutants by adsorption on activated carbon prepared from agricultural waste. Chem. Eng. J..

[77] Khan M.N., Sarwar A. (2007). Determination of Points of Zero Charge of Natural and Treated Adsorbents. Surf. Rev. Lett..

[78] Fiol N., Villaescusa I. (2009). Determination of sorbent point zero charge: Usefulness in sorption studies. Environ. Chem. Lett..

[79] Ţucureanu V., Matei A., Avram A.M. (2016). FTIR Spectroscopy for Carbon Family Study. Crit. Rev. Anal. Chem..

[80] Tsai W.T., Chang C.Y., Lee S.L., Wang S.Y. (2001). Thermogravimetric analysis of corn cob impregnated with zinc chloride for preparation of activated carbon. J. Therm. Anal. Calorim..

[81] Nielsen L., Biggs M.J., Skinner W., Bandosz T.J. (2014). The effects of activated carbon surface features on the reactive adsorption of carbamazepine and sulfamethoxazole. Carbon.

[82] Svahn O., Björklund E. (2015). Thermal stability assessment of antibiotics in moderate temperature and subcritical water using a pressurized dynamic flow-through system. Int. J. Innov. Appl. Sci..

[83] Nguyen D.T., Tran H.N., Juang R.-S., Dat N.D., Tomul F., Ivanets A., Woo S.H., Hosseini-Bandegharaei A., Nguyen V.P., Chao H.-P. (2020). Adsorption process and mechanism of acetaminophen onto commercial activated carbon. J. Environ. Chem. Eng..

[84] Jendrzejewska I., Goryczka T., Pietrasik E., Klimontko J., Jampílek J. (2020). X-ray and Thermal Analysis of Selected Drugs Containing Acetaminophen. Molecules.

[85] Tortet L., Ligner E., Blanluet W., Noguez P., Marichal C., Schäf O., Paillaud J.-L. (2017). Adsorptive elimination of paracetamol from physiological solutions: Interaction with MFI-type zeolite. Micropor. Mesopor. Mater..

[86] Jasper E.E., Ajibola V.O., Onwuka J.C. (2020). Nonlinear regression analysis of the sorption of crystal violet and methylene blue from aqueous solutions onto an agro-waste derived activated carbon. Appl. Water Sci..

[87] Cheng T., Li J., Ma X., Zhou L., Wu H., Yang L. (2021). The adsorption properties of microporous activated carbon prepared from pistachio nut shell for low-concentration VOCs under low-medium temperatures. Environ. Sci. Pollut. Res..

[88] Gil A., Taoufik N., Garcia A., Korili S. (2019). Comparative removal of emerging contaminants from aqueous solution by adsorption on an activated carbon. Environ. Technol..

[89] Sellaoui L., Gómez-Avilés A., Dhaouadi F., Bedia J., Bonilla-Petriciolet A., Rtimi S., Belver C. (2022). Adsorption of emerging pollutants on lignin-based activated carbon: Analysis of adsorption mechanism via characterization, kinetics and equilibrium studies. Chem. Eng. J..

[90] Lee S., Kim N., Park D. (2023). Insights into the Adsorption Performance of Emerging Contaminants on Granular Activated Carbon. Separations.

[91] Diniz V., Rath G., Rath S., Araújo L., Cunha D. (2021). Competitive kinetics of adsorption onto activated carbon for emerging contaminants with contrasting physicochemical properties. Environ. Sci. Pollut. Res..

[92] Kozyatnyk I., Oesterle P., Wurzer C., Mašek O., Jansson S. (2021). Removal of contaminants of emerging concern from multi-component systems using carbon dioxide activated biochar from lignocellulosic feedstocks. Bioresour. Technol..

[93] Motaghi H., Arabkhani P., Parvinnia M., Asfaram A. (2022). Simultaneous adsorption of cobalt ions, azo dye, and imidacloprid pesticide on the magnetic chitosan/activated carbon@UiO-66 bio-nanocomposite: Optimization, mechanisms, regeneration, and application. Sep. Purif. Technol..

[94] Diniz V., Cunha D., Rath S. (2022). Adsorption of recalcitrant contaminants of emerging concern onto activated carbon: A laboratory and pilot-scale study. J. Environ. Manag..

[95] Álvarez-Torrellas S., Rodríguez A., Ovejero G., García J. (2016). Comparative adsorption performance of ibuprofen and tetracycline from aqueous solution by carbonaceous materials. Chem. Eng. J..

[96] Fotsop C.G., Lieb A., Scheffler F. (2025). Core-shell material based on ion-exchange-assisted growth of ZIFs on chamfered-edged zeolite crystals for N_2_/CO_2_ adsorption: Modeling and mechanism. J. Environ. Chem. Eng..

[97] de Ridder D.J., Verliefde A.R., Heijman S.G., Verberk J.Q., Rietveld L.C., van der Aa L.T., Amy G.L., van Dijk J.C. (2011). Influence of natural organic matter on equilibrium adsorption of neutral and charged pharmaceuticals onto activated carbon. Water Sci. Technol..

[98] Margot J., Kienle C., Magnet A., Weil M., Rossi L., de Alencastro L.F., Abegglen C., Thonney D., Chèvre N., Schärer M. (2013). Treatment of micropollutants in municipal wastewater: Ozone or powdered activated carbon?. Sci. Total Environ..

[99] Altmann J., Ruhl A.S., Zietzschmann F., Jekel M. (2014). Direct comparison of ozonation and adsorption onto powdered activated carbon for micropollutant removal in advanced wastewater treatment. Water Res..

[100] Mailler R., Gasperi J., Coquet Y., Deshayes S., Zedek S., Cren-Olivé C., Cartiser N., Eudes V., Bressy A., Caupos E. (2015). Study of a large scale powdered activated carbon pilot: Removals of a wide range of emerging and priority micropollutants from wastewater treatment plant effluents. Water Res..

[101] PubChem. https://pubchem.ncbi.nlm.nih.gov/.

[102] Boreen A.L., Arnold W.A., McNeill K. (2004). Photochemical Fate of Sulfa Drugs in the Aquatic Environment:  Sulfa Drugs Containing Five-Membered Heterocyclic Groups. Environ. Sci. Technol..

[103] Steiner T. (2002). The Hydrogen Bond in the Solid State. Angew. Chem. Int. Ed..

[104] Caron G., Ermondi G. (2016). Molecular Descriptors for Polarity: The Need for Going Beyond Polar Surface Area. Future Med. Chem..

[105] Ertl P., Rohde B., Selzer P. (2000). Fast Calculation of Molecular Polar Surface Area as a Sum of Fragment-Based Contributions and Its Application to the Prediction of Drug Transport Properties. J. Med. Chem..

[106] Lopez-Ramon M.V., Stoeckli F., Moreno-Castilla C., Carrasco-Marin F. (1999). On the characterization of acidic and basic surface sites on carbons by various techniques. Carbon.

[107] Frisch M.J., Trucks G.W., Schlegel H.B., Scuseria G.E., Robb M.A., Cheeseman J.R., Scalmani G., Barone V., Petersson G.A., Nakatsuji H. (2016). Gaussian 16, Revision A.03.

[108] Peverati R., Truhlar D.G. (2012). Screened-exchange density functionals with broad accuracy for chemistry and solid-state physics. Phys. Chem. Chem. Phys..

[109] Weigend F., Ahlrichs R. (2005). Balanced basis sets of split valence, triple zeta valence and quadruple zeta valence quality for H to Rn: Design and assessment of accuracy. Phys. Chem. Chem. Phys..

[110] Marenich A.V., Cramer C.J., Truhlar D.G. (2009). Universal solvation model based on solute electron density and a continuum model of the solvent defined by the bulk dielectric constant and atomic surface tensions. J. Phys. Chem. B.

[111] Dennington R., Keith T.A., Millam J.M. (2016). GaussView.

[112] Lu T., Chen F. (2012). Multiwfn: A Multifunctional Wavefunction Analyzer. J. Comput. Chem..

[113] Lu T., Chen Q. (2022). Realization of Conceptual Density Functional Theory and Information-Theoretic Approach in Multiwfn Program. Conceptual Density Functional Theory.

[114] Humphrey W., Dalke A., Schulten K. (1996). VMD: Visual molecular dynamics. J. Mol. Graph..

