# Supplementary material for: Sustainable Remediation of Pharmaceuticals Using Crop-Residue-Derived Carbons: Bridging Multi-Component Adsorption and DFT Perspectives
Source: Molecules. 2026 Mar 31;31(7):1162. doi: 10.3390/molecules31071162 (PMC13074987; doi:10.3390/molecules31071162)
Supplement: Supplementary file 1 [file molecules-31-01162-s001.zip › molecules-4195892-supplementary.pdf]

Sustainable Remediation of Pharmaceuticals using Crop Resi-due-Derived Carbons: Bridging Multi-component Adsorption and DFT Perspectives

Assel A. Kurtebayeva[1], Silvia Álvarez-Torrellas[2*], Juan García[2], Helder T. Gomes[3], Juan M. Garrido-Zoido[4], M. Victoria Gil[4], Seitzhan A. Orynbayev[1], Marzhan S. Kalmakhanova[1*]

[1]*M.Kh. Dulaty Taraz University, Department of Chemistry and Chemical Technology, 080012 Taraz, Kazakhstan*
[2]*Catalysis and Separation Processes Group, Chemical Engineering and Materials Department, Faculty of Chemistry, Complutense University, Avda. Complutense s/n, 28040 Madrid, Spain*
[3]*CIMO, LA SusTEC, Instituto Politécnico de Bragança, Campus de Santa Apolónia, 5300-253 Bragança, Portugal*
[4]*IACYS-Green Chemistry and Sustainable Development Unit, Department of Organic and Inorganic Chemistry, Faculty of Sciences, University of Extremadura, 06006 Badajoz, Spain*

**Corresponding authors: satorrellas@ucm.es; marjanseitovna@mail.ru.*

DFT calculation parameters

The energy gap (E_{gap}), Fermi level (EFL), ionization potential (IP), electron affinity (EA), global hardness (η), global softness (S), chemical potential (μ) and electrophilicity index (ω), were determined using the following equations:

$$E_{gap} = E_{LUMO} - E_{HOMO} \quad \text{(S1)}$$

$$E_{FL} = E_{HOMO} + \frac{E_{LUMO} - E_{HOMO}}{2} \quad \text{(S2)}$$

$$IP \approx -E_{HOMO} \quad \text{(S3)}$$

$$EA \approx -E_{LUMO} \quad \text{(S4)}$$

$$\eta = \frac{E_{LUMO} - E_{HOMO}}{2} \quad \text{(S5)}$$

$$S = \frac{1}{\eta} \quad \text{(S6)}$$

$$\mu = \frac{E_{LUMO} + E_{HOMO}}{2} \quad \text{(S7)}$$

$$\omega = \frac{\mu^2}{2 \cdot \eta} \quad \text{(S8)}$$

Figures

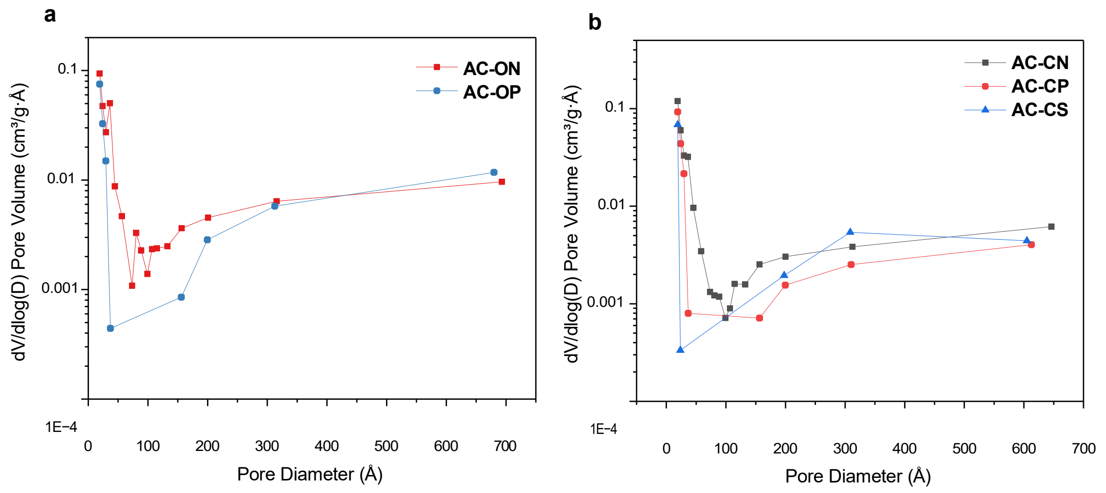

Figure S1. Pores size distributions of the activated carbons obtained from **(a)** onion waste **(b)** corn cob.

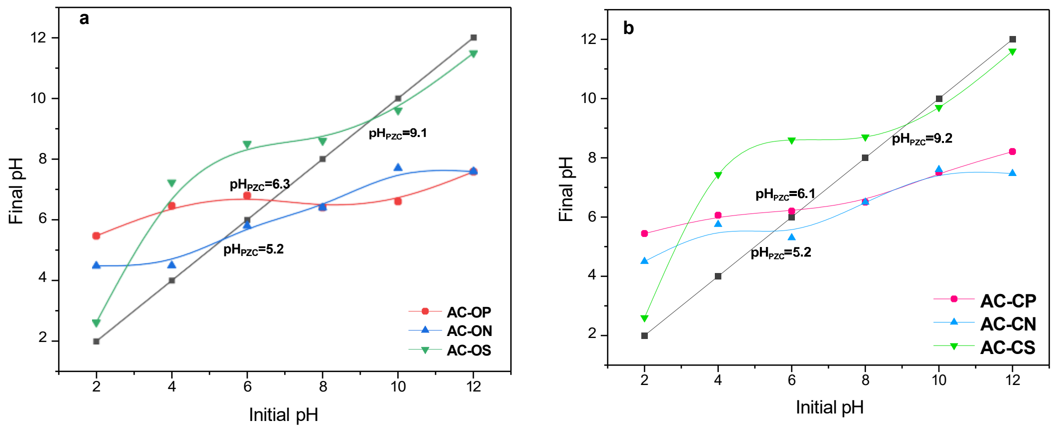

Figure S2. pH_{PZC} values of the activated carbons obtained from **(a)** onion waste **(b)** corn cob.

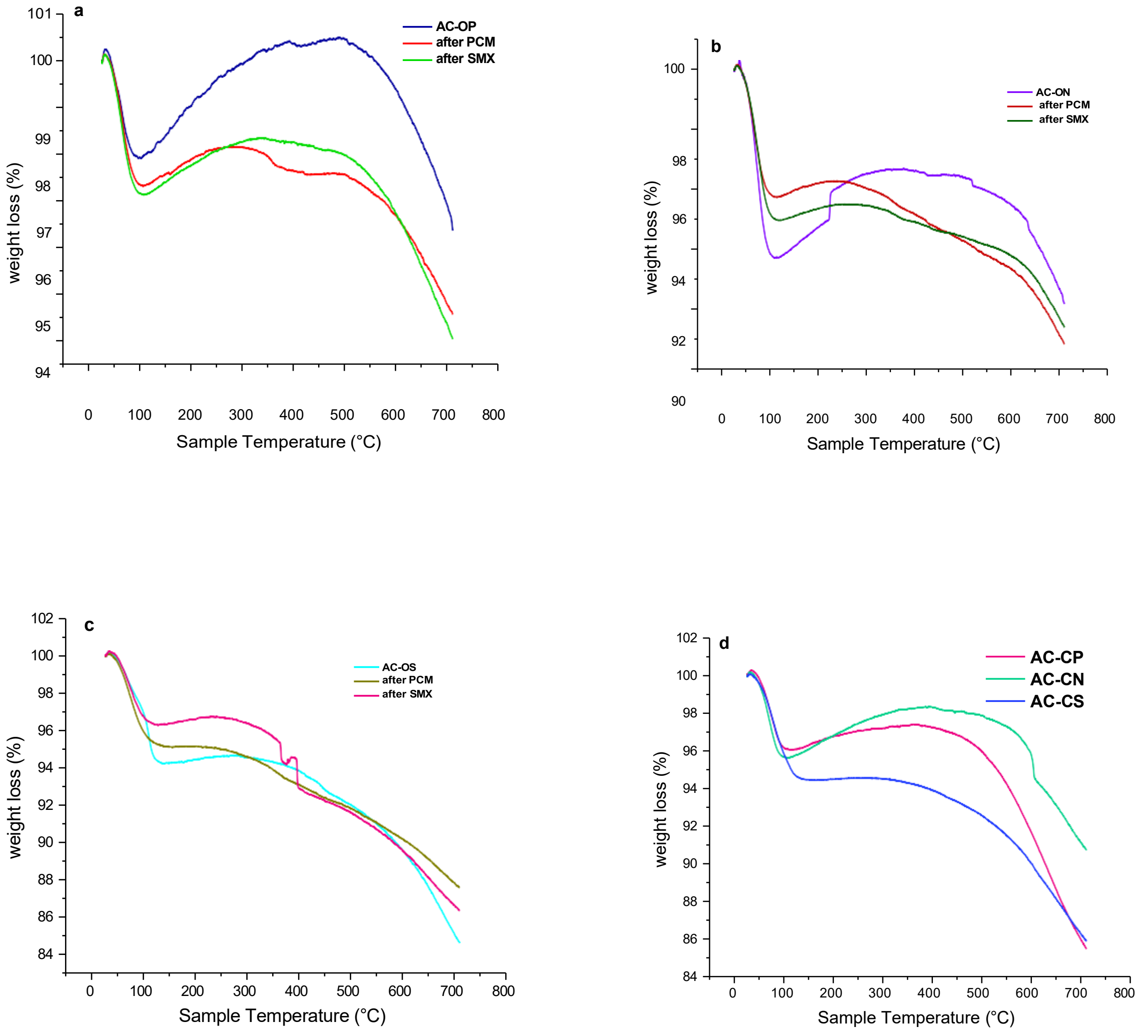

Figure 3S. TG profiles of the activated carbons prepared from
(a) onion wastes (AC-OP) and after adsorption PCM, SMX
(b) onion wastes (AC-ON) and after adsorption PCM, SMX
(c) onion wastes (AC-OS) and after adsorption PCM, SMX
(d) corncob wastes (AC-CP, AC-CN, AC-CS)

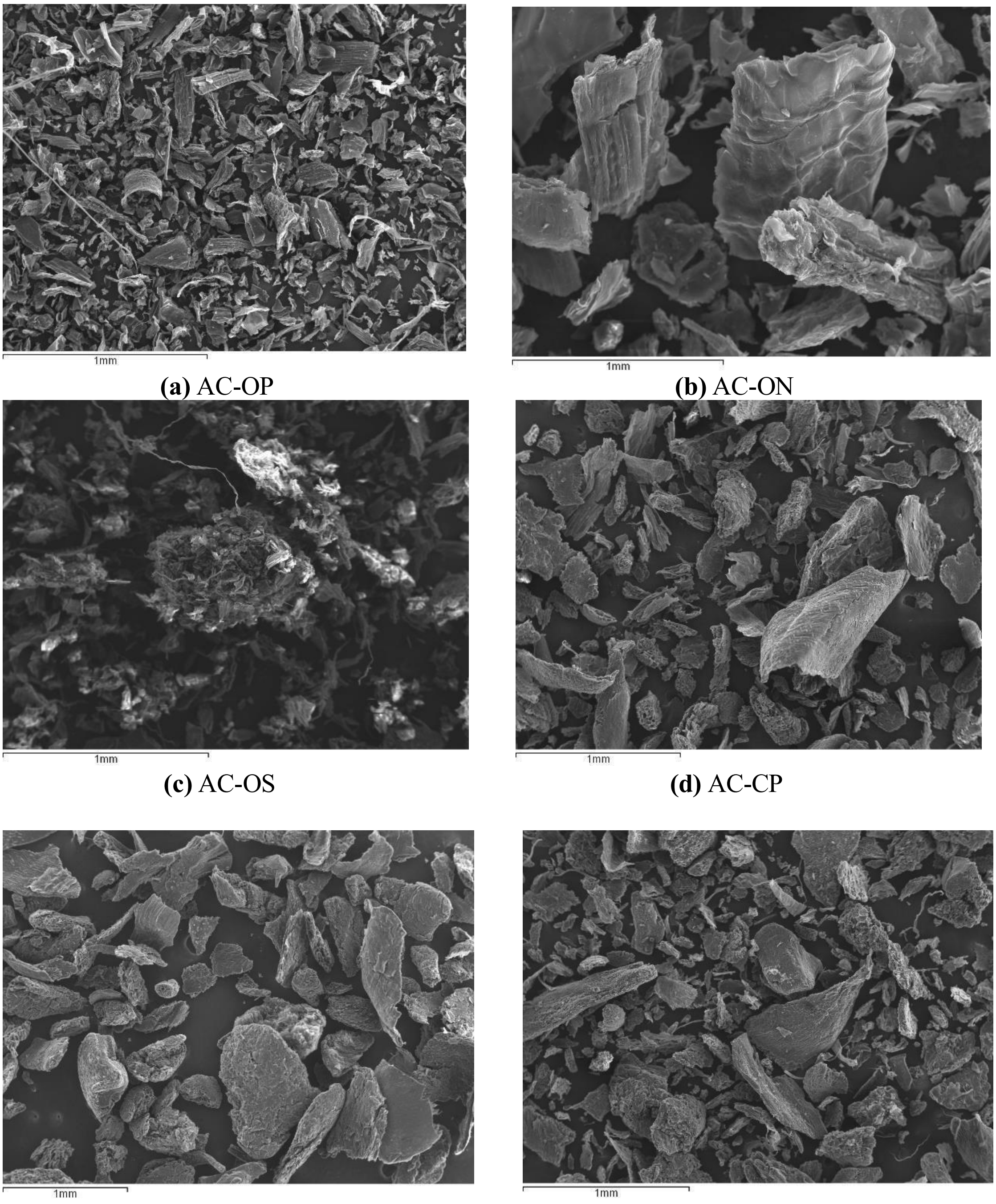

(a) AC-OP **(b)** AC-ON

(c) AC-OS **(d)** AC-CP

(e) AC-CN **(f)** AC-CS

Figure S4. SEM micrographs of the synthesized activated carbons.

Tables

Table S1. Elemental analysis of the synthesized activated carbons.

Elemental compound	AC-OP	AC-ON	AC-OS	AC-CP	AC-CN	AC-CS
**C (wt.%)**	76.14	73.70	64.75	77.49	76.08	76.75
**H (wt.%)**	2.62	2.58	2.30	2.68	2.60	2.70
**N (wt.%)**	1.18	2.45	1.46	0.77	1.93	1.00
**S (wt.%)**	0.09	0.06	0.06	0.06	0.06	0.03
**O (wt.%)**	19.97	21.21	31.43	19.00	19.33	19.52

Table S2. Physico-chemical parameters of the WWTP effluent tested.

Parameter	
**pH**	7.9
**Salinity (ppt)**	0.75
**TDS (mg/L)**	714
**Conductivity (µS/cm)**	1,445
**Total N (mg/L)**	40.73
**TOC (mg/L)**	1,106

Table S3. Cartesian coordinates for optimized structure of paracetamol (MN12-SX/def2-TZVP, SMD solvation: water)

ATOM TYPE	x	y	z
C	-2.58814000	-0.10698600	0.01553200
C	-1.72118500	-1.17449200	-0.16282600
C	-0.35360000	-0.96930400	-0.22727900
C	0.16625700	0.31591300	-0.10147900
C	-0.71166200	1.38247600	0.06817300
C	-2.07668200	1.17915000	0.12353700
H	-2.12654400	-2.17740800	-0.26000600
H	0.30358300	-1.81368600	-0.38652400
H	-0.31322100	2.39045300	0.15637900
H	-2.75266300	2.02005600	0.25582200
O	-3.92289300	-0.36834500	0.07287400
H	-4.41355900	0.45407100	0.18725900
N	1.53802700	0.61805800	-0.17508300
C	2.59051400	-0.18868800	0.06836500
O	2.47955900	-1.37377000	0.37018300
C	3.93829300	0.45656000	-0.06062500
H	3.88749400	1.52902100	-0.24850000
H	4.50239300	0.27210000	0.85625000
H	4.47679400	-0.02593600	-0.88054900
H	1.75343000	1.59407200	-0.33938400

Table S4. Cartesian coordinates for optimized structure of sulfamethoxazole (MN12-SX/def2-TZVP, SMD solvation: water)

ATOM TYPE	x	y	z
C	3.04375600	-0.44300500	-1.00638600
C	3.06492700	-1.49536200	-0.07443700
C	2.16059200	-1.46371400	0.99898600
C	1.26952500	-0.42509600	1.13531400
C	1.26625100	0.61234300	0.20524200
C	2.15463100	0.59402700	-0.87011800
N	3.91579200	-2.54107700	-0.23341500
S	0.11902900	1.90161800	0.33526400
N	-1.13045500	1.56720700	-0.68510400
O	-0.42373900	1.90350600	1.65555900
O	0.67402200	3.10227400	-0.20486700
C	-1.84673500	0.38361500	-0.57066800
C	-2.23364400	-0.30455200	0.60283400
C	-2.95193300	-1.34898700	0.12742200
O	-2.99726800	-1.29668800	-1.20788400
N	-2.27988200	-0.19601500	-1.65771700
C	-3.66097100	-2.46534000	0.77692300
H	3.74557600	-0.45745100	-1.83583100
H	2.17600300	-2.26976700	1.72727700
H	0.57911100	-0.40575800	1.97387600
H	2.15152300	1.40455600	-1.59435200
H	4.07530200	-3.14645100	0.55934200
H	4.69968200	-2.42232600	-0.85941600
H	-0.92928800	1.84269500	-1.64595800
H	-2.02939000	-0.05680000	1.63131100
H	-4.72369400	-2.44456300	0.51972400
H	-3.25473700	-3.42368300	0.44132700
H	-3.55524700	-2.39346600	1.85899000

Table S5. Cartesian coordinates for optimized structure of sulfamethoxazole anion (MN12-SX/def2-TZVP, SMD solvation: water)

ATOM TYPE	x	y	z
C	2.77276300	-0.53256400	-1.27319000
C	3.19732600	-1.30216700	-0.18101100
C	2.60312200	-1.07735300	1.06466900
C	1.61831700	-0.11996000	1.21303700
C	1.20393700	0.63646600	0.12533900
C	1.78950400	0.41918200	-1.11948100
N	4.13362100	-2.29206900	-0.34725800
S	-0.05265500	1.86568300	0.27299100
N	-1.20771300	1.50970700	-0.71349300
O	-0.44083000	1.86850900	1.67137600
O	0.51968700	3.11149800	-0.19102900
C	-1.92023700	0.36661500	-0.51370300
C	-1.84735100	-0.62690400	0.51568200
C	-2.79539500	-1.52854100	0.18697500
O	-3.41262800	-1.15078100	-0.93604700
N	-2.85740300	0.05584700	-1.39072500
C	-3.25456000	-2.78691800	0.80683000
H	3.23081000	-0.69581900	-2.24540600
H	2.92614500	-1.66546000	1.91984900
H	1.16669800	0.04402900	2.18720700
H	1.47143100	1.00790300	-1.97694600
H	4.62033200	-2.58649900	0.48926100
H	4.73806200	-2.19753600	-1.15292600
H	-1.19573500	-0.65323500	1.37439400
H	-4.31630100	-2.72543200	1.06299500
H	-3.12875200	-3.62534000	0.11547700
H	-2.68414500	-2.98886500	1.71328100

Table S6. Energetic data for optimized structures of paracetamol (PCM), sulfamethoxazole (SMX) and sulfamethoxazole anion (SMX^-) at MN12-SX/def2-TZVP level, with SMD solvation model; solvent = water.

Molecule	Electronic energy (kcal/mol)	Free energy (kcal/mol)	Entropy (cal/(mol·K))	Enthalpy (kcal/mol)
**PCM**	−323359.09	−323281.36	100.56	−323251.38
**SMX**	−737616.31	−737511.75	130.47	−737472.85
**SMX^-**	−737326.47	−737228.86	125.89	−737191.33

Table S7. Electronic parameters and chemical descriptors of PCM, SMX and SMX^- calculated at MN12-SX/def2-TZVP level considering water solvation (SMD method). Values in eV, except for S (eV^{-1}).

Descriptor*	PCM	SMX	SMX^-
E_{HOMO}	−5.81	−5.99	−5.60
E_{LUMO}	−0.74	−1.11	−0.70
E_{gap}	5.07	4.88	4,90
E_{FL}	−3.28	−3.55	−3.15
IP	5.81	5.99	5.60
EA	0.74	1.11	0.70
η	2.54	2.44	2.45
S	0.39	0.41	0.41
μ	−3.28	−3.55	−3.15
ω	2.12	2.58	2.03

**Energy gap (E_{gap}), Fermi level (E_{FL}), ionization potential (IP), electron affinity (EA), global hardness (η), global softness (S), chemical potential (μ) and electrophilicity index (ω).*
